# Cohort profile: the Genetics of Glucose regulation in Gestation and Growth (Gen3G) – a prospective prebirth cohort of mother–child pairs in Sherbrooke, Canada, 3-year and 5-year follow-up visits

**DOI:** 10.1136/bmjopen-2024-093434

**Published:** 2025-03-22

**Authors:** Amélie Taschereau, Myriam Doyon, Mélina Arguin, Catherine Allard, Véronique Desgagné, Anne-Marie Cote, Éric Massé, Pierre-Étienne Jacques, Patrice Perron, Marie-France Hivert, Luigi Bouchard

**Affiliations:** 1 Biochimie et génomique fonctionnelle, University of Sherbrooke, Sherbrooke, Quebec, Canada; 2 Centre de Recherche du Centre Hospitalier Universitaire de Sherbrooke, Sherbrooke, Quebec, Canada; 3 Department of Medicine, University of Sherbrooke, Sherbrooke, Quebec, Canada; 4 Department of Biology, Université de Sherbrooke, Sherbrooke, Quebec, Canada; 5 Harvard Medical School, Boston, Massachusetts, USA; 6 Department of Population Medicine, Harvard Pilgrim Health Care Institute, Boston, Massachusetts, USA; 7 Department of Biochemistry, Université de Sherbrooke, Sherbrooke, Quebec, Canada

**Keywords:** EPIDEMIOLOGY, GENETICS, Follow-Up Studies, Pregnancy, Obesity

## Abstract

**Abstract:**

**Purpose:**

Initiated in 2010, the Genetics of Glucose regulation in Gestation and Growth (Gen3G) prospective cohort investigates the pathophysiology of impaired glycaemic regulation in pregnancy and evaluates its impact on both the mothers and her offspring health trajectory. Follow-up visits 3 and 5 years after delivery aimed to investigate pregnancy-related risk factors such as maternal obesity and gestational hyperglycaemia in relation to the mother’s metabolic health after pregnancy, and with offspring health outcomes such as risk of obesity and neurodevelopmental problems in early childhood. We also investigated molecular mechanisms involved in the fetal programming of these later health outcomes.

**Participants:**

Of the 1024 women originally recruited in the first trimester of pregnancy, we have targeted the 854 who had complete glucose tolerance test data and the 724 newborns who provided placenta and/or cord blood samples for follow-up recruitment. Of these, 695 mother–child dyads agreed to be contacted for the prospective follow-up visits. 448 and 521 mother–child dyads completed the research visits at 3 and 5 years after delivery respectively.

**Findings to date:**

At both visits, we collected the mother’s and child’s medical history, lifestyle (using validated questionnaires), sociodemographic status, anthropometric measurements, mother’s blood samples, child’s saliva samples and growth charts. At the 5-year-old visit, we additionally collected the mother’s and child’s urine and stool samples and the child’s blood samples; we performed a 75 g oral glucose tolerance test in the mothers and assessed the body composition in children using dual-energy X-ray absorptiometry. Using the Gen3G rich longitudinal data set, we have enhanced the understanding of the pathophysiology and characterisation of the heterogeneity of gestational diabetes mellitus, and we have shown that gestational hyperglycaemia and insulin resistance are associated with offspring epigenetics (DNA methylation) variations in the placenta, cord blood and blood at 5 years of age, as well as with offspring anthropometric, metabolic and neurodevelopmental outcomes in early childhood.

**Future plans:**

We are currently conducting a prospective follow-up of mothers and their children 12 years after delivery to study how prenatal and early-life metabolic factors may programme childhood adiposity and obesogenic dietary behaviours. This follow-up should be completed by the end of 2026.

**STRENGTHS AND LIMITATIONS OF THIS STUDY**

- Prospective design from the beginning of pregnancy to 3 and 5 years after delivery, allowing detailed, standardised collection of biological samples and anthropometric measurements to provide information on numerous potential covariates in epidemiological analyses, while also allowing longitudinal analysis.
- Body composition has been assessed in the majority of 5-year-old children using dual-energy X-ray absorptiometry technology, the gold standard for assessing body composition.
- Our biobank contains a variety of biological samples, including blood, hair (child only), stool and urine samples from both mothers and children, as well as a large amount of omics data, which will allow future studies to apply artificial intelligence and deep learning algorithms.
- The sample size is relatively small for genetic association studies but reasonable for other omics studies in the context of refined phenotype and longitudinal follow-up of participants.
- The sample population includes most people of French–Canadian descent, which limits the generalisability of our findings.

## Introduction

 Pregnancy imposes significant stresses on maternal metabolism to support fetal growth and development.^1^ Pregnancy-specific conditions may reveal an increased susceptibility to cardiometabolic diseases later in life for the mother while exposing the offspring to an increased risk of chronic diseases. Gestational diabetes mellitus (GDM) is defined as a type of diabetes first diagnosed during pregnancy. It is the most common pregnancy metabolic complication,^2^ affecting approximately 14% of pregnancies worldwide.^3^ GDM is associated with a 7 to 10-fold higher risk of developing type 2 diabetes (T2D) for affected women,^4^ whereas fetal exposure to GDM predisposes the offspring to the development of obesity^5^ and adverse neurodevelopmental outcomes in their later life.^6^ According to the Developmental Origin of Health and Diseases (DOHaD) hypothesis, derived from Barker’s hypothesis,^7^ these predispositions might be programmed by epigenetic modifications,^8^ but the pathophysiological mechanisms supporting the DOHaD remain largely unknown.

The Genetics of Glucose regulation in Gestation and Growth (Gen3G) prospective cohort was initiated in 2010 to study biological, environmental and genetic determinants of glucose regulation during pregnancy and the impacts of glucose metabolism dysregulation in pregnancy on future maternal and offspring health. The pregnancy phase of the Gen3G cohort has been described previously. Since then, both the mothers and their offspring were reassessed 3 and 5 years after delivery. The objectives of the follow-up visits among Gen3G participants were twofold. First, we aimed to investigate the mother’s metabolic health a few years after delivery. Second, with this longitudinal follow-up, we aimed to expand our understanding of gestational metabolic exposures associated with obesity and neurodevelopmental problems in early childhood. Using omics analyses, we aimed to identify potential underlying molecular mechanisms of DOHaD.

The Gen3G cohort has many unique characteristics including recruitment in the first weeks of pregnancy, comprehensive metabolic phenotyping during pregnancy including screening for prepregnancy diabetes (based on A1c and 50 g glucose challenge test) and GDM (with 75 g-oral glucose tolerance test (OGTT) and collection of additional maternal plasma samples), multiple metabolic biomarkers (eg, insulin, lipids, adipokines) and the collection of cord blood and placenta samples (all described in prior cohort profile^9^). This updated cohort profile describes the two follow-up visits of mother–child dyads at 3 and 5 years after delivery and the rich Gen3G data now available including multiple blood biomarkers, body composition assessment in children (using dual-energy X-ray absorptiometry (DXA)) and the availability of multiomic data (genome-wide DNA methylation analysis, transcriptomics, whole genome sequencing, metabolomics, microbiome analyses, etc). Gen3G provides a unique opportunity to study the DOHaD hypothesis and its associated mechanisms. Gen3G has contributed to the development of scientific knowledge in four major domains: (1) pathophysiology of glucose regulation in pregnancy and heterogeneity of GDM; (2) epidemiological analyses supporting the DOHaD hypothesis; (3) potential mechanisms implicated in DOHaD, specifically epigenetic investigations, and (4) contributions to large genetic and epigenetic consortia related to pregnancy and early life metabolic traits. We provided an overview of our contributions in the discussion of this cohort profile. By presenting this cohort profile, we aimed to share the methods and procedures we have developed for monitoring mothers and children from the first weeks of pregnancy and now up to 5 years after birth, and to encourage collaboration with other cohorts and the use of the Gen3G Cohort data and biosamples.

### Cohort description

Gen3G is a prospective pregnancy and birth cohort that recruited 1024 pregnant women in the first trimester of pregnancy in Sherbrooke, Canada, between January 2010 and June 2013. Briefly, all pregnant women who were receiving their prenatal care at the Centre Hospitalier et Universitaire de Sherbrooke (CHUS) or at a CHUS-affiliated centre and who were planning to deliver at the CHUS were invited to participate in Gen3G. Gen3G participants show similar characteristics as compared with the pregnant women followed at the CHUS at the time of recruitment in terms of age, parity, prepregnancy body mass index (BMI) and ethnic background.^9^ We have diagnosed GDM using a 75 g-OGTT in the 24–28 weeks using the criteria (fasting plasma glucose≥5.1 mmol/L and/or 1-hour plasma glucose≥10.0 mmol/L and/or 2-hour plasma glucose≥8.5 mmol/L) recommended by the International Association of Diabetes and Pregnancy Study Group.^10^ At delivery, we had 854 women still enrolled in the study and had completed the 75 g-OGTT in the late second trimester.

**Figure 1:**
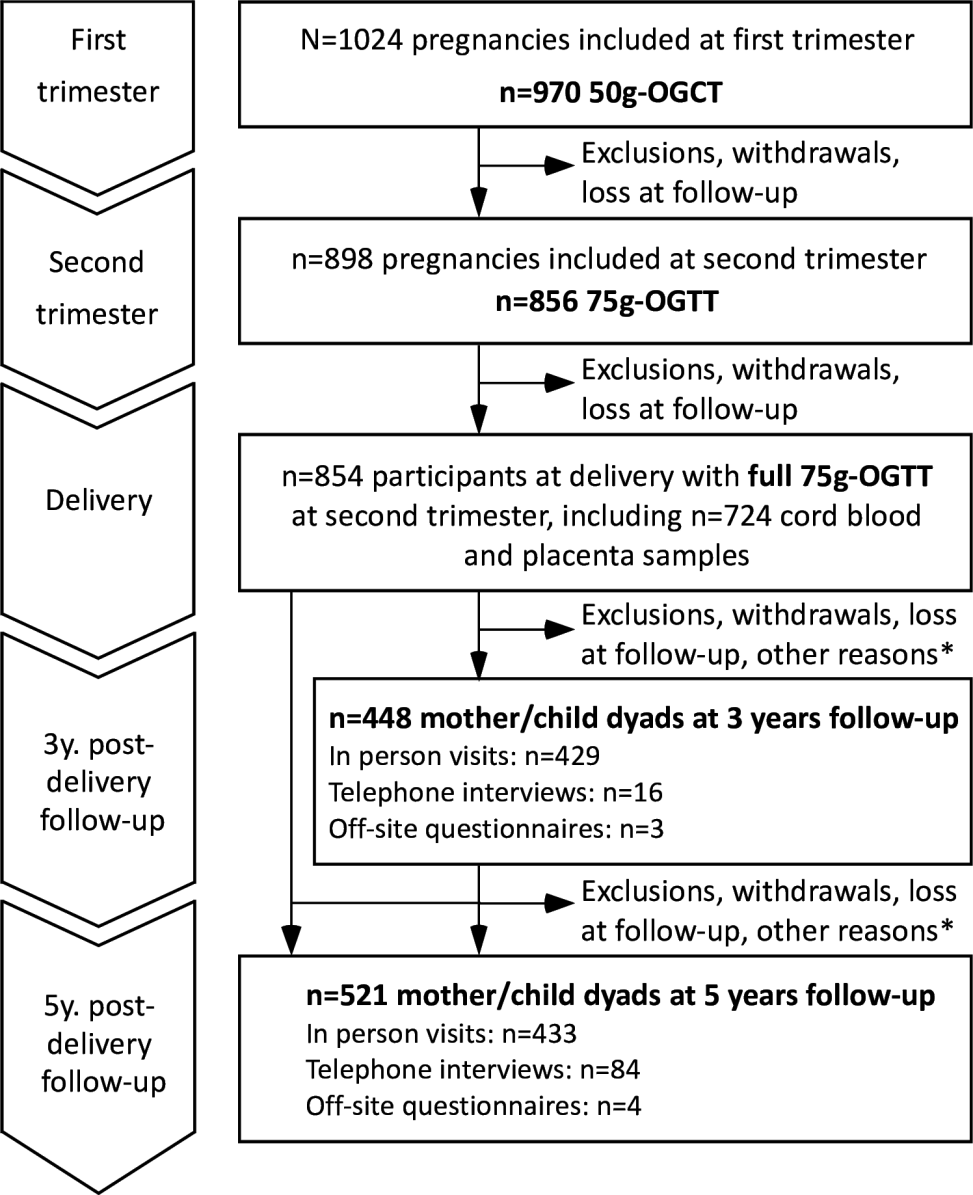
Flow chart illustrating the number of participants enrolled and active in the Gen3G cohort from initial recruitment at first trimester of pregnancy to prospective follow-up visits during childhood. *Participants were excluded if the child’s age was out of the window of follow-up. Other reasons for not attending the 3 and/or 5-year follow-up visits are: unable to contact (outreached without answers, contact information not valid), never show up for the scheduled visit, participant declined this follow-up but agreed to be recontacted for future assessments. Gen3G, Genetics of Glucose regulation in Gestation and Growth; OGCT, oral glucose challenge test; OGTT, oral glucose tolerance test.

Among the 854 women eligible for follow-ups, we were able to collect placenta and/or cord blood samples from 724 of their newborns.^9^ Of these, 695 accepted to be recontacted for prospective follow-up visits. Eligible mothers were contacted by phone and invited to participate, with their children, at the 3-year and 5-year follow-up visits. 448 and 521 mother–child dyads participated in the 3-year and 5-year follow-up visits, respectively (figure 1). Table 1 shows the characteristics of mothers and children in the Gen3G cohort who were recruited at the 3-year and 5-year follow-up visits compared with mothers and children who were excluded, withdrawn or lost to follow-up.

**Table 1:** Baseline characteristics of mothers and children in the Gen3G cohort contributing data at the 3-year and 5-year follow-up visits compared with those who were excluded, withdrawn or lost to follow-up

Characteristics	3-year visit			5-year visit		
	Participants with data collected (n=448)	Participants not included in the follow-up (n=576)	P value	Participants with data collected (n=521)	Participants not included in the follow-up (n=503)	P value
N (%) or median (IQR) or mean±SD						
**Mothers**						
Ethnicity (European descent)	430 (96)	553 (97)	0.74	503 (97)	480 (96)	0.74
N available	448	573		521	500	
Age at first trimester of pregnancy	28.2±4.6	28.6±4.3	0.15	28.1±4.7	28.5±4.2	0.15
N available	448	576		521	503	
Primigravid (yes)	157 (35)	196 (34)	0.74	179 (34)	174 (35)	0.95
N available	448	576		521	503	
Positive family history of diabetes (yes)	90 (20)	100 (17)	0.29	101 (19)	89 (18)	0.52
N available	448	574		521	501	
Prepregnancy maternal BMI	23.6 (21.2–27.5)	23.0 (20.8–27.3)	0.15	23.3 (21.1–27.3)	23.0 (20.9–27.4)	0.57
N available	441	573		513	501	
Smoking status at first trimester (yes)	36 (8)	78 (14)	**0.007**	44 (9)	70 (14)	**0.007**
N available	445	574		517	501	
GDM diagnosis (yes)*Among GDM, number treated with insulin	7 (11)17 (4)	25 (6)11 (2)	**0.01**0.57	53 (10)16 (3)	19 (5)12 (2)	**0.01**0.85
N available	436	420		508	348	
**Offspring**						
Sex (girls)	209 (47)	217 (50)	0.42	248 (48)	183 (51)	0.78
N available	448	430		521	357	
Child’s gestational age at delivery	39.5 (38.6–40.3)	39.7 (38.9–40.3)	0.28	39.6 (38.7–40.4)	39.6 (38.7–40.3)	0.96
N available	446	430		520	356	
Weight at birth (kg)	3.4±0.5	3.4±0.5	0.95	3.4±0.5	3.4±0.5	0.87
N available	446	429		520	355	

p-P value was determined with Fisher’s exact test for categorical data (ethnicity, gravidity, familial diabetes history, maternal smoking status, GDM diagnosis, child’s sex), non-parametric Mann-Whitney U test for continuous data not normally distributed (pre-pregnancy maternal BMI and child gestational age) and Student’s Tt-test for continuous variables normally distributed (first-trimester maternal age and child weight at birth). Significant P value<0.05 are in bold.Abbreviations:

* By IADPSG criteria: 75 g-OGTT with fasting, 1 and 2-hour plasma glucose. If fasting plasma glucose ≥5.1 mmol/L and/or 1-hour plasma glucose ≥10.0 mmol/L and/or 2-hour plasma glucose ≥8.5 mmol/L.

BMI, body mass index; GDM, gestational diabetes mellitus; Gen3G, Genetics of Glucose regulation in Gestation and Growth

### Study procedures

We collected data and samples from both mothers and children at each follow-up visit. Mothers were required to fast for 12 hours before both visits. If mothers accepted the genetic component of the study (n=492), we also collected the buffy coats (peripheral white blood cells) and monocytes obtained from their blood sample collections. We collected buffy coats for the genetic component of the study because they are easy to collect and provide a high quantity and quality of DNA.^11^

At the 3-year follow-up visit, we collected mothers’ medical history and lifestyle habits (nutrition and physical activity using an adapted version of the Canadian Community Health Survey (CCHS), alcohol consumption, smoking status, sleep duration and sun exposure) as well as information on their sociodemographic status (including educational level and current marital status). Using standardised procedures (all described in the data collection section), we collected the mother’s anthropometric measurements (height, weight, waist circumference), vital signs and fasting blood samples. We asked the mothers about their child’s medical history and lifestyle habits (nutrition and physical activity using an adapted version of the CCHS, sleep duration, secondhand smoke exposure and screen time). Mothers completed a questionnaire about their child’s strengths and weaknesses in various activities of daily living (Strength and Difficulties (SDQ) validated questionnaire for neurobehavioural development assessment). Again in a standardised manner, we collected the child’s anthropometric measurements (height, weight, waist and hip circumferences, and skinfold thicknesses), vital signs and a saliva sample. We also collected general information about the child’s father (ethnicity, age, weight, height, diabetes diagnosis and health status). We asked mothers’ consent to contact the child’s physician to access his/her growth chart. For the 3-year visit, among the 695 mother–child dyads attempted to recontact; 22 (3.1%) were excluded because the child had reached age>3.5 years (cut-off point set to avoid overburdening mother–child dyads by requiring them to attend two visits too close together); 103 (14.5%) mothers were unreachable or did not return our calls; 27 (3.8%) declined to participate in the 3-year follow-up visit (but accepted to be recontacted at a later time point), and 95 (13.3%) withdrew from the Gen3G study. Our sample thus included 448 (62.9%) mother–child dyads at the 3-year follow-up visit; 429 (95.8%) completed the in-person visit at the CHUS Research Centre (CRCHUS); 16 (3.6%) completed questionnaires by phone, and 3 (0.7%) returned completed at-home questionnaires by mail.

At the 5-year follow-up visit, we updated mothers and the child’s medical history, lifestyle habits and sociodemographic status and repeated mothers and the child’s anthropometric measurements with standardised protocols. Additionally, mothers completed a questionnaire about their feelings and behaviours experienced during the past 7 days (Center for Epidemiologic Studies Depression Scale (CES-D Scale)). In addition to the SDQ questionnaire, mothers completed a questionnaire about their child’s temperament (Achenbach System of Empirically Based Assessment (ASEBA) questionnaire). If mothers consented, children completed a full-body composition analysis by DXA and fasted for 8 hours prior to the visit if willing to provide blood samples. If mother accepted the genetic component of the study for children (n=484), we also collected the buffy coats (peripheral white blood cells) obtained from their blood sample collections. We also invited mothers to perform a 75 g-OGTT during which we collected blood samples at fasting, 1-hour and 2-hour post ingestion. In addition to blood (mother and child: plasma and buffy coat), we collected the child’s saliva (same collection kit used at the 3-year visit), urine, hair and stool samples and maternal urine and stool samples (same collection kit used for children). Families collected stool samples at home with provided kits, which were returned by mail. Finally, we collected updated information on the child’s father (ethnicity, age, weight, height, diabetes diagnosis and health status). Among the 600 mother–child dyads still eligible and with available contact information, 13 (2.1%) mothers were unreachable or never returned our calls; 12 (1.9%) mother–child dyads did not show up for the scheduled visit; 1 (0.2%) mother declined to participate in the 5-year follow-up visit but agreed to be recontacted for future assessments, and 70 (11.4%) mothers withdrew from the study. For the 5-year follow-up, 17 women with GDM or low birth weight, for whom we did not collect a biological sample at birth and who were excluded from the 3-year follow-up initially, were invited to the 5-year follow-up visit. Our final sample at the 5-year follow-up visit included 521 mother–child dyads; 433 (83.1%) of which completed the in-person visit at the CRCHUS; 84 (16.1%) completed questionnaires by phone, and 4 (0.8%) returned questionnaires completed at home by mail. At both 3-year and 5-year follow-up visits, we assessed maternal pregnancy status, and pregnant women did not undergo anthropometric measurements, blood sampling and the 75 g OGTT (5-year visit).

Table 1 presents the characteristics of Gen3G participants (mothers and offspring) compared with those who were excluded, withdrawn or lost to follow-up, for both childhood follow-up visits. No significant baseline demographic differences between groups were observed. The proportions of women who were primigravid and had a positive familial history of diabetes were similar between groups. There were no significant group differences for the prepregnancy maternal BMI and offspring’s birth weight. For both follow-up visits, positive smoking status at the first trimester of pregnancy was slightly less common in women who remained in the study, whereas positive diagnosis of GDM was slightly more common in women who remained in the study.

## Data collection

### Questionnaires (3-year and 5-year)

Trained research staff administered standardised and validated questionnaires at both visits. They collected details on participants’ medical history, sociodemographic status, lifestyle habits, diet and physical and sedentary activities. The full list of data collected is described in box 1. Table 2 and table 3 provides a general overview of the characteristics of the mothers and their children.

Box 1 **List of data domains collected from staff-administered questionnaires and/or electronic medical data records**

Season of visit

**Mothers:**

- Age
- Ethnicity
- Medical history (including number of antibiotic cycles since birth)
- Medication in use at the time of the visit (including natural products, vitamin and mineral supplementation)
- Level of education, type of employment, marital status
- Alcool, tabacco use (current and daily consumption, or never, prior and cessation date) and sleep duration
- Questionnaire on sun exposure
- Center for Epidemiologic Studies Depression (CES-D) scale (depres- sive state) (only completed at the 5-year follow-up visit)
- Derived from the CCHS questionnaire:

  Diet (summary of weekly consumption of fish and sugar-sweetened beverages (including juices, sodas, etc.), daily consumption of fruits, vegetables and dairy products, breakfast habits, restaurant visits and type of water consumed.

Physical activities:

- Commuting and daily living (including type of commuting, choice of stairs vs elevators
- Last 3 months of leisure physical activities (metabolic equivalent of tasks (METs) estimation)
- Weekly sedentary recreational activities time accorded to computer, video game, television and reading

**Offpsring:**

- Age
- Sexe
- Ethnicity
- Gestational age at birth
- Birth weight
- Maternal breastfeeding (yes/no and duration)
- Growth chart (N accessible)
- Medical history (including number of antibiotic cycles since birth)
- Medication in use at the time of the visit (including natural products, vitamin and mineral supplementation)
- Lifestyle habits (secondhand smoke exposure, sleep duration)
- Strength and Difficulties (SDQ) Questionnaire version Pa 3/4
- Achenbach System of Empirically Based Assessment (ASEBA) Questionnaire (1.5–5 years) Canadian (Québec) version (only completed at the 5-year follow-up visit)
- Derived from the CCHS questionnaire:

  Diet (type of breastfeeding, age of introduction of solid foods, summary of weekly consumption of fish and sweetened beverages (including juices, sodas, etc), daily consumption of fruits and vegetables, dairy products, breakfast habits, restaurant visits and type of water consumed).

Physical activities:

- Commuting and daily living (including type of commuting, choice of stairs vs elevators
- Last 3 months of leisure physical activities (metabolic equivalent of tasks (METs) estimation)
- Weekly sedentary recreational activities time accorded to computer, video game, television and reading

**Fathers:**

- Age
- Ethnicity
- Height
- Weight
- Body mass index
- Diabetes diagnosis
- Other health conditions

**Table 2:** Characteristics of Gen3G mothers and fathers during the 3-year and 5-year follow-up visits

Parameters	3-year visit		5-year visit	
	Total valid n*	Value N (%) or median (IQR) or mean±SD	Total valid n*	Value N (%) or median (IQR) or mean±SD
**Mothers**				
Age (years)	448	32.5±4.3	520	34.5±4.3
Ethnicity (European descent)	447	429 (96)	519	500 (96)
Subsequent pregnancy (at least one)	446	232 (52)	518	299 (58)
Pregnancy status (positive)	448	42 (9)	521	21 (4)
Medical history (self-reported as diagnosis)				
Asthma	444	46 (10)	518	46 (9)
Medical history (self-reported as diagnosis)	446	59 (13)	518	70 (14)
Urinary problems	446	9 (2)	517	15 (3)
T2D diagnosis	447	0 (0)	516	1 (<0.01)
Allergies	447	166 (37)	518	211 (41)
Gastric reflux	446	52 (12)	517	78 (15)
Mental health problems	447	62 (14)	515	101 (20)
*Sociodemographic questionnaire*				
Level of education	446		519	
High school		33 (7)		37 (7)
Professional degree		91 (20)		116 (22)
College		128 (29)		130 (25)
University—baccalaureate		122 (28)		159 (31)
University—higher degree		72 (16)		77 (15)
Current marital status	446		518	
Common law or married		345 (77)		451 (87)
Single parent		55 (12)		49 (9)
Divorced		46 (10)		18 (3)
Lifestyle habits				
Alcohol consumers (currently consuming)	447	345 (77)	519	452 (87)
Smoking (currently smoking)	448	45 (10)	517	60 (12)
Sleep duration (hours per night)	432	7.3± 1.2	509	7.3±1.1
Nutrition (derived from the CCHS questionnaire)				
Fruits (times per day)	445	2.0 (1.5–3.0)	519	2.0 (1.0–3.0)
Vegetables (times per day)	447	2.0 (2.0–3.0)	515	2.0 (2.0–3.0)
Fish (times per week)	447	1.0 (0.9–2.0)	519	1.0 (0.5–2.0)
Dairy products (times per day)	445	2.0 (1.0–3.0)	517	2.0 (1.0–2.5)
Sugar-sweetened beverages (times per week)	447	3.0 (1.0–7.0)	518	2.0 (0.5–7.0)
Breakfast consumption (every day or most days)	447	369 (83)	518	415 (80)
Restaurant consumption (times per month)	446	4.0 (2.0–5.0)	519	4.0 (2.0–5.0)
Physical activity questionnaire (derived from the CCHS questionnaire)				
Energy spent (kcal/kg/day)	447	1.59 (0.76–2.64)	518	1.76 (0.96–3.15)
Depressive state (CES-D Scale) (score—possible range: 0–60)	n/a	n/a	485	7 (3–11)
**Fathers** **†**				
Age (years)	441	34.7±4.9	512	36.7±4.9
Ethnicity (European descent)	447	432 (97)	512	494 (96)
Weight (kg)	423	86.36 (77.27–95.45)	502	84.09 (77.27–95.45)
Height (m)	414	1.78 (1.73–1.83)	506	1.78 (1.73–1.83)
BMI (kg/m2)‡	411	26.6 (24.3–29.9)	500	26.6 (24.4–29.8)
Diabetes diagnosis (yes)	440	10 (2)	512	19 (4)
Other health conditions (N available)	448	434 (97)	521	500 (96)

* This n indicates the maximum number of participants that contributed data for a specific variable at a specific time point.

† Reported by the mother at both visits.

‡ Based on the mother’s reported values.

BMI, body mass index; CCHS, Canadian Community Health Survey; Gen3GGenetics of Glucose regulation in Gestation and GrowthScale, Center for Epidemiologic Studies Depression ScaleT2Dtype 2 diabetes

**Table 3:** Characteristics of Gen3G children during the 3-year and 5-year follow-up visits

Parameters	3-year visit		5-year visit	
	Total valid n*	Value N (%) or median (IQR) or mean±SD	Total valid n*	Value N (%) or median (IQR) or mean±SD
Sex (girls)	448	209 (47)	521	248 (48)
Ethnicity (European descent)	448	425 (95)	521	494 (95)
Gestational age at birth (weeks)	446	39.5 (38.6–40.3)	520	39.6 (38.4–40.4)
Birth weight (kg)	446	3.40±0.49	520	3.40±0.48
Maternal breastfeeding (yes)	429	353 (82)	519	439 (85)
Breastfeeding duration (months)	429	6 (1–11)	519	6 (1–11)
Mixed feeding (yes)	316	123 (39)	515	242 (47)
Age at which solid food was introduced (months)	444	5.5 (4.0–6.0)	515	5.5 (4.0–6.0)
Age at the childhood visits (months)	448	40.6±2.9	521	64.8±4.4
Growth chart (N accessible)	448	431 (96)	521	499 (96)
Medical diagnosis (Mother reported)				
Asthma	446	28 (6)	520	29 (6)
Allergies	446	69 (15)	519	96 (18)
Mental health problems	447	5 (1)	518	21 (4)
Lifestyle habits				
Secondhand smoke exposed (yes)	446	26 (6)	519	43 (8)
Sleep duration for napping (hours per day)	448	1.8 (1.5–2.0)	514	0.5 (0.0–1.1)
Sleep duration during the night (hours per night)	448	10.0 (10.0–11.0)	520	10.0 (9.7–10.8)
Nutrition (derived from the CCHS questionnaire)—mother reported				
Fruits (serving per day)	447	3.0 (2.0–4.0)	517	3.0 (2.0–3.5)
Vegetables (serving per day)	446	2.0 (2.0–3.0)	517	2.0 (2.0–3.0)
Fish (serving per week)	447	1.0 (1.0–2.0)	520	1.0 (0.5–2.0)
Dairy products (serving per day)	443	3.5 (2.5–4.0)	516	3.0 (2.0–4.0)
Sweetened beverages (serving per week)	445	7.0 (1.0–7.0)	520	3.5 (1.0–7.0)
Breakfast consumption (every day or most day)	448	443 (99)	520	518 (99)
Restaurant consumption (time per month	448	2 (1–4)	519	2 (1–4)
Physical activity (derived from the CCHS questionnaire) (N available)	448	448 (100)	521	520 (99)
SDQ Questionnaire (score—possible range: 0–50)	442	8 (5–12)	517	7 (5–11)
ASEBA Behavioural Questionnaire (score—possible range: 0–198)	n/a	n/a	485	27 (17–40)

* This n indicates the maximum number of participants that contributed data for a specific variable at a specific time point.

ASEBAAchenbach System of Empirically Based AssessmentCCHSCanadian Community Health SurveyGen3GGenetics of Glucose regulation in Gestation and GrowthSDQStrength and Difficulties Questionnaire

### Blood and other biological samples collected (3-year and 5-year)

We collected fasting plasma from EDTA tubes and Cell Preparation Tube (CPT) (BD Vacutainer® CPT™ Cell Preparation Tube with Sodium Heparin), and if the genetic component was accepted, we collected buffy coat from EDTA tubes and monocytes from CPT from blood samples in mothers at both visits, and in children at 5 years only (if children consented to blood collection). At the 5-year visit, if the mother consented to the OGTT, we collected an additional 8 mL of maternal plasma (two additional 4 mL lavender EDTA tubes) at each time point of the OGTT (fasting, 1 hour and 2 hours post-OGTT). Using American Diabetes Association (ADA) criteria,^12^ we identified 70 additional women with prediabetes (ADA criteria: fasting plasma glucose 5.6 mmol/L to 6.9 mmol/L and/or 2-hour plasma glucose 7.8 mmol/L to 11.0 mmol/L and/or glycated haemoglobin (HbA1c) 5.7% to 6.4%) and four additional women with T2D (ADA criteria: fasting plasma glucose≥7 mmol/L and/or 2-hour plasma glucose≥11.1 mmol/L and/or HbA1c≥6.5%) at the 3-year and 5-year follow-up visits. Child’s saliva was collected at both visits using the Super•SAL Saliva Collection Kit (Oasis diagnostics, Washington, USA) according to the manufacturer’s instructions. At the 5-year visit, we collected child’s hair and urine samples and mother’s urine samples. Families also collected stool samples at home using OMNIgene•GUT|OMR-200 (DNA Genotek Ontario, Canada) and returned by mail. Consent to collect stool and urine samples was given independently. Table 4 summarises the biological samples collected. Tables5  6 summarise the biological data collected and the blood biomarkers measured in Gen3G mothers and children respectively.

**Table 4:** Biological samples collected during the 3-year and 5-year follow-up visits

Sample type	Number of participants who accepted biobanking and provided the listed sample			
	Mothers		Offspring	
	3-year	5-year	3-year	5-year
Blood*				
Fasting plasma	355	390	n/a	315
Fasting plasma CPT	343	383	n/a	297
75 g-OGTT plasma				
Plasma T0-fasting	n/a	390	n/a	n/a
Plasma T1 hour	n/a	326	n/a	n/a
Plasma T2 hours	n/a	326	n/a	n/a
Genetic component				
Buffy coat	334	393	n/a	325
Monocytes	329	377	n/a	306
Saliva	n/a	n/a	128	383
Microbiome stool	n/a	244	n/a	228
Urine	n/a	313	n/a	338
Hair	n/a	n/a	n/a	370

* In addition to the fasting blood samples reported here, we collected non-fasting blood samples from 2 additional mothers at 3 years postpartumpost-delivery, 4 additional mothers at 5 years postpartum,-delivery and 10 additional children at 5 years postpartum-delivery.Abbreviations:

CPT, Cell Preparation Tube; OGTT, oral glucose tolerance test

**Table 5:** Biological data from Gen3G mothers collected during the 3-year and 5-year follow-up visits

Parameters	3-year visit		5-year visit	
	Total valid N*	ValueN (%) or median (IQR) or mean±SD	Total valid N*	ValueN (%) or median (IQR) or mean±SD
*Blood biochemistry* *†*				
Glycaemic regulation				
Fasting insulin (pg/mL)	367	666.8 (329.1–1729.5)	408	618.66 (287.44–1536.00)
Fasting C-peptide (pg/mL)	369	896.3 (671.8–1192.0)	411	1023.0 (789.7–1288.0)
Fasting glycaemia (pg/mL)	372	4.5 (4.3–4.8)	408	4.7 (4.4–5.0)
HbA1c (%)	373	5.3 (5.1–5.4)	411	5.2 (5.0–5.4)
75 g-OGTT				
1-hour glucose (pg/mL)	n/a	n/a	381	5.7 (4.4–7.1)
2-hour glucose (pg/mL)	n/a	n/a	381	5.1 (4.4–6.0)
1-hour insulin (pg/mL)	n/a	n/a	339	2627 (1616–4008)
2-hour insulin (pg/mL)	n/a	n/a	338	2043 (1357–3392)
Lipid profile				
Total cholesterol (mmol/L)	374	4.32±0.81	410	4.40±0.83
HDL cholesterol (mmol/L)	374	1.52±0.36	410	1.59±0.41
LDL cholesterol (mmol/L)	374	2.42±0.75	410	2.38±0.74
ApoA (mmol/L)	n/a	n/a	391	1.60±0.29
ApoB (mmol/L)	n/a	n/a	391	0.84±0.20
Triglycerides (mmol/L)	374	0.74 (0.54–1.06)	410	0.82 (0.62–1.20)
Others				
Calcium (mmol/L)	360	2.28±0.09	393	2.29±0.09
Phosphates (mmol/L)	358	0.97±0.14	393	1.00±0.14
Parathyroid hormone (mmol/L)	359	4.0 (3.30–5.15)	391	4.30 (3.45–5.30)
Creatinine (µmol/L)	n/a	n/a	409	63.63±8.89
Leptin (pg/mL)	369	5575 (2594–10 157)	411	9047.0 (4306.5–16 658.0)
Adiponectin (pg/mL)	369	49.95 (28.28–78.48)	411	25.12 (14.51–45.66)
TNFα (pg/mL)	369	3.50±1.27	411	4.24±1.57
PAI-1 (ng/mL)	369	9.54 (6.00–16.84)	411	11.86 (6.74–19.31)
MCP1 (pg/mL	369	64.74 (52.69–79.98)	411	76.71 (63.97–91.30)
Urine sample (yes)	n/a	n/a	521	331 (64)
Stool sample (yes)	n/a	n/a	521	252 (48)
Anthropometry and vital signs				
Weight (kg)	395	67.00 (57.80–79.40)	500	67.10 (59.09–80.05)
Height (m)‡	395	1.65±0.06	500	1.65±0.06
BMI (kg/m^2^)	395	24.4 (21.6–29.1)	500	24.7 (21.9–29.0)
Total fat (%)	378	32.8 (26.4–39.0)	412	33.5 (27.7–39.8)
Waist circumference (cm)	366	86.0 (78.3–97.0)	413	87.9 (80.0–99.8)
Systolic blood pressure (mm Hg)	376	111.6±9.2	414	110.5±9.5
Diastolic blood pressure (mm Hg)	376	72.2±7.0	414	72.4±6.5
Heart rate (bpm)	374	72±10	414	73±10

* Indicates the maximum number of participants that contributed data for a specific variable at a specific time point.

† Mothers were required to fast for 12 hours before blood was drawn.

‡ Reported pregnancy measurement.

A, apolipoprotein A; B, apolipoprotein B; BMI, body mass index; Gen3GGenetics of Glucose regulation in Gestation and GrowthHbA1cglycated haemoglobinHDL, high-density lipoprotein; LDL, low-density lipoprotein; MCP1, monocyte chemoattractant protein-1; OGTT, oral glucose tolerance test; PAI-1, plasminogen activator inhibitor-1; TNFα, tumour necrosis factor-α

**Table 6:** Biological data from Gen3G children collected during the 3-year and 5-year follow-up visits

Parameters	3-year visit		5-year visit	
	Total valid n*	Value N (%) or median (IQR) or mean±SD	Total valid n*	Value N (%) or median (IQR) or mean±SD
*Blood biochemistry* *†*				
Glycaemic regulation				
Fasting insulin (pg/ml)	n/a	n/a	322	332.98 (175.62–879.80)
Fasting C-peptide (pg/ml)	n/a	n/a	331	420.05 (245.74–548.14)
Fasting glycaemia (pg/ml)	n/a	n/a	340	4.5±0.4
HbA1c (%)	n/a	n/a	343	5.2±0.3
Lipid profile				
Total cholesterol (mmol/L)	n/a	n/a	349	3.91±0.60
HDL cholesterol (mmol/L)	n/a	n/a	349	1.53±0.33
LDL cholesterol (mmol/L)	n/a	n/a	349	2.12±0.55
ApoA (mmol/L)	n/a	n/a	332	1.44±0.20
ApoB (mmol/L)	n/a	n/a	332	0.74±0.15
Triglycerides (mmol/L)	n/a	n/a	349	0.54 (0.43–0.67)
Others				
Calcium (mmol/L)	n/a	n/a	333	2.43±0.09
Phosphates (mmol/L)	n/a	n/a	332	1.47 (1.39–1.54)
Parathyroid hormone (mmol/L)	n/a	n/a	330	3.1 (2.6–3.9)
Creatinine (µmol/L)	n/a	n/a	350	31.05±4.85
Leptin (pg/mL)	n/a	n/a	331	444.79 (247.48–868.54)
Adiponectin (pg/mL)	n/a	n/a	341	38.19 (21.28–57.93)
TNFα (pg/mL)	n/a	n/a	331	5.42±1.96
PAI-1 (ng/mL)	n/a	n/a	341	6.01 (4.39–9.47)
CP1 (pg/mL)	n/a	n/a	331	67.34 (54.09–82.85)
Saliva sample collected (yes)	448	143 (32)‡	521	410 (79)
Urine sample (yes)	n/a	n/a	521	361 (69)
Stool sample (yes)	n/a	n/a	521	241 (46)
Hair sample (yes)	n/a	n/a	521	393 (75)
Anthropometry and vital signs				
Weight (kg)	442	15.12 (14.00–16.51)	512	19.20 (17.70–20.96)
Height (m)	432	0.97±0.04	505	1.11±0.05
BMI Z-score	431	0.55 ±0.94	504	0.25±1.01
Waist circumference (cm)	421	51.0 (49.0–53.0)	428	53.5 (51.9–55.7)
Hip circumference (cm)	418	53.8 (51.5–56.5)	428	57.9 (55.6–60.1)
Sum of skinfolds (mm)§	403	29.3 (25.5–34.4)	423	29.3 (24.7–35.0)
Arm length (cm)	419	19.7±1.4	427	23.4±1.5
Arm circumference (cm)	423	16.6 (15.8–17.4)	426	17.3 (16.5–18.2)
Systolic blood pressure (mm Hg)	385	97.4±7.7	423	100.2±6.8
Diastolic blood pressure (mm Hg)	385	63.9±6.0	423	63.7±5.0
Heart rate (bpm)	380	103±11	423	93±11

* Indicates the maximum number of participants that contributed data for a specific variable at a specific time point.

† Children were required to fast for 8 hours before blood was drawn.

‡ Measure added late for the 3-year follow-up visit.

§ Calculated from measured skinfold thickness (triceps, biceps, subscapular and suprailiac folds).

A, apolipoprotein A; B, apolipoprotein B; BMI, body mass index; Gen3GGenetics of Glucose regulation in Gestation and GrowthHbA1c, glycated haemoglobin; HDL, high-density lipoprotein; LDL, low-density lipoprotein; MCP1, monocyte chemoattractant protein-1; PAI-1, plasminogen activator inhibitor-1; TNFα, tumour necrosis factor-α

### Anthropometry and vital signs (3-year and 5-year)

Trained research staff completed measures using standardised procedures. We measured the child’s weight (kg) and height (cm) barefoot and in light clothing using a calibrated electronic scale (Rice Lake Weighing system, Rice Lake, Wisconsin, USA) and a wall stadiometer (Seca, Hamburg, Germany), respectively. We calculated BMI as weight divided by square height (kg/m^2^) and BMI z-scores using either the WHO Anthro or AnthroPlus Softwares for children aged under 5 years and for children aged 5 years and over, respectively.^13^ We measured the maternal and child’s waist circumference (cm) once, to the nearest 0.1 cm, using a flexible measuring tape above the top of the iliac crest.^14^ We also measured the child’s hip circumference (cm) once to the nearest 0.1 cm, using a flexible measuring tape at the widest part of the pelvis. We measured systolic and diastolic blood pressures (mm Hg) and heart rates in the sitting position after resting for 5 min. Systolic and diastolic blood pressures and heart rate measures were performed twice or thrice if the difference between the two measures was greater than 10%, and the average of all measures taken was recorded.

In children, we measured arm length (cm) and arm circumference (cm) to the nearest 0.1 cm using a flexible measuring tape. Measures were performed twice and thrice if the difference between the first two measures was greater than 10%. A trained research assistant measured skinfold thicknesses (mm) twice on the right side of the body using standard procedures to the nearest 0.5 mm at biceps, triceps and subscapular and suprailiac areas using a skinfold calliper (AMG Medical, Mont-Royal, QC, Canada). A third measurement was done if the difference between the first two measures was greater than 10%.

We estimated body fat percentage in mothers by bioimpedance using a standing foot-to-foot scale (TBF-300A; Tanita; coefficient of variation (CV): 2.1%^15^).

### DXA (5-year)

On mother’s consent, we assessed the child’s whole body composition using DXA scan (Horizon DXA system, Hologic, Marlborough, Massachusetts, USA), the gold standard for assessing body composition in children.^16^ After mother and child assent, a trained research assistant asked the children to remain still during the scan and helped to place their limbs in the correct position on the instrument. In 130 cases where the children moved slightly during the scan, resulting in asymmetrical results, the scans were carefully reviewed by two research staff and the data from the limb that had not moved (fat mass and lean mass) was duplicated and applied to the limb suspected of having moved. The research team (including principal investigators) reviewed all scans that were difficult to assess to make decisions on limb duplication or exclusion of images.^17^ We used Hologic software (V.5.5.3.1) to define body regions and to calculate body fat percentages. Table 7 shows sex-stratified DXA scan measures for children assessed at the 5-year follow-up visit, given the known sex difference in body composition.

**Table 7:** DXA scan measures at 5 years of age reported by sex

Parameters	Girls n=185ValueMedian (IQR) or mean±SD	Boys n=197ValueMedian (IQR) or mean±SD
Total fat mass (%)	32.91±4.46	28.47±3.26
Total fat mass (g)	5851.0 (5271.2–6878.9)	5297.2 (4684.1–5917.8)
Trunk fat mass (%)	28.0 (25.1–31.3)	23.5 (21.5–25.9)
Trunk fat mass (g)	2171.5 (1899.7–2548.6)	1896.3 (1688.1–2156.6)
Total lean mass (%)	63.71 (61.06–66.47)	68.30 (66.62–70.41)
Total lean mass (g)	11 957.65±1738.37	12 970.84±1570.93
Total mass (g)	18 117.6 (16 983.1–20 087.8)	18 873.7 (17 515.2–20 314.8)

DXA, dual-energy X-ray absorptiometry

## Handling of biological specimen

Blood glucose, lipid profile, calcium, phosphates, creatinine, apolipoprotein A1 and apolipoprotein B were measured on fresh samples immediately on collection at the CHUS biochemistry-accredited clinical laboratory. Blood samples processed to obtain DNA, RNA and plasma were promptly centrifuged at 2500 × g for 10 min at 4°C. Plasma aliquots of ∼500 µl were wrapped in parafilm to avoid evaporation and stored at −80°C for future RNA extraction and analysis or biobanking (table 4). Buffy coats were stored at −80°C for future DNA extraction and analyses. After collection, children’s saliva samples were wrapped in parafilm to avoid evaporation and stored at −80°C. Urine samples were aliquoted in 1.5 mL tubes and stored at −80°C within 2 hours after collection.

At the 5-year follow-up only, stool samples were collected at home and returned by mail at the CRCHUS. The samples were stored at −80°C until gut microbiota characterisation. Hair samples were collected from children for future cortisol analysis as previously described.^18^

### Analyses of selected biomarkers

Analyses of selected biomarkers were performed in our endocrinology research laboratory. The aliquots of plasma were thawed on ice and centrifuged at 6000 × g for 10 min at 4℃. Analytes were measured by multiplexed particle-based flow cytometric assay with Luminex technology (EMD Millipore) according to the manufacturer’s recommendations and protocols. Two panels were used: HMHEMAG-34K (insulin, C-peptide, monocyte chemoattractant protein-1, leptin and tumour necrosis factor-a (TNF-α)) and HADK1MAG-61K (adiponectin and plasminogen activator inhibitor-1 (PAI-1)). Intra-assay and interassay CVs were, respectively<1.63% and <28.62% for all analytes of HMHEMAG-34K, and<1.04% and <46.89% for both analytes of HAD1MAG-61K. For interassay %CV calculation, we used three internal controls that were replicated on every assay. The value of %CV was obtained by calculating the mean of the mean of each internal control %CV. For adiponectin and PAI-1 interassay %CV, the concentrations of our three internal controls were outside the range of linearity of the standard curves, hence the %CV higher than recommended by the manufacturer. See online supplemental table 1 for more details on each analyte.

### Patient and public involvement

Patients and the public were not involved in any way in the production of this research.

### Findings to date

So far, the scientific contributions of Gen3G are numerous (see online supplemental table 2 for a list of all Gen3G publications to date) and diverse, spanning four major domains. First, Gen3G contributed to improving the understanding of the pathophysiology and characterisation of the heterogeneity of GDM. Briefly, we described GDM subtypes based on differences in insulin sensitivity and secretion indexes, and how they are associated with different outcomes at delivery (C-section, macrosomia, neonatal hypoglycaemia)^19^ as well as differences in maternal blood lipid profiles.^20^ We also demonstrated the associations between maternal circulating levels of adiponectin, TNFa and vitamin D and the risk of GDM and/or insulin sensitivity in pregnancy.^21^,^23^ We investigated sleep duration in pregnancy in relation to maternal blood glucose.^24^ More recently, we characterised the plasma circulating microtranscriptome (microRNA) profile in pregnancy^25^ and identified subsets of first-trimester microRNAs associated with fasting glycaemia at the second trimester of pregnancy,^26^ predictive of lower insulin sensitivity in pregnancy^27^ and increased risk of GDM.^28^ The discovery of microRNAs opens a new era for disease screening and diagnostics, including for GDM and gestational hypertension. Our recent genome-wide RNA sequencing analyses of placenta samples revealed that a deficit in placental IGFBP1 likely contributes to the pathophysiology of GDM and higher insulin resistance in pregnancy.^29^ Our studies identified many biomarkers that could allow identification of high-risk women by the end of the first trimester of pregnancy to predict who are the pregnant people developing GDM, opening the door for precision prevention of pregnancy complications.

Second, Gen3G contributed to demonstrating the associations between prenatal exposures related to maternal ‘dysmetabolism’ and various adverse offspring outcomes. Gen3G provided evidence that mid/late excessive gestational weight gain and maternal leptin levels are associated with increased birth size.^30 31^ We have demonstrated associations between maternal metabolic profile in pregnancy (specifically insulin sensitivity) and child’s cardiac function,^32^ adiposity at 3 and 5 years old, and metabolic or inflammatory biomarkers including metabolomic profiling in children’s circulating plasma at 5 years old.^1733^,^37^ We have also studied the gut microbiome in early childhood and showed that prepregnancy BMI, as well as breastfeeding and complementary (solid) food introduction are associated with altered gut microbiota composition.^38 39^

Third, Gen3G allowed the investigation of mechanisms by which maternal adiposity, blood glucose and GDM may influence offspring anthropometric, metabolic and neurodevelopment in the context of DOHaD through fetal epigenetic programming.^40^,^44^ We have identified DNA methylation variations in the placenta, cord blood and blood at 5 years of age associated with exposure to maternal metabolic/glycaemic indexes as well as associations with children’s anthropometric, metabolic and neurodevelopmental phenotypes in early life.^4244^,^55^ In support of the overall concept of fetal metabolic programming and DOHaD, some of the genes containing the identified loci are known to regulate energy metabolism (*LRP1*,^42^ *CACNA1D*^55^ *, LRP1B*^55^ *, LPL*^54^ *, KCNQ1*,^52^ *DLGAP2*,^52^ *FAM3C*^51^ *, URGCP*^50^), adipose tissue development (*BRD2*^55^ *, PPARGC1a*^44^) and neurodevelopment (*LRP1B*,^55^ *NEGR1*^53^ *, DLGAP2*^52^ *, TFAP2E*^51^ *, CRHBP*^46^). Despite findings from Gen3G and many other prebirth cohorts, additional studies are still needed to confirm that fetal epigenetic programming is at the heart of the DOHaD concept, and to identify which loci are mechanistically involved in long-term risk of metabolic diseases across the life course.

Finally, Gen3G contributed to genetic studies and meta-analyses from international consortia (Pregnancy And Childhood Epigenetics, Early Growth Genetics (EGG) and GENetics of Diabetes in Pregnancy (GenDIP)) to demonstrate that GDM shares genetic risk with T2D,^56^,^67^ which broadens the scientific impact of Gen3G.

Gen3G is not the only longitudinal mother–child cohort that started in pregnancy and prospectively follows up participants in childhood. Some other well-known cohorts with similar aims and data include Project Viva, Avon Longitudinal Study of Parents and Children (ALSPAC), Generation R, Aboriginal Birth Cohort (ABC), Family Atherosclerosis Monitoring in Early Life (FAMILY) and SouTh Asian biRth cohorT (START). Gen3G set itself apart by its detailed phenotyping overall and glucose physiology in particular, its rich biobank and the assessment of body fat using DEXA scan in children at 5 years of age. Our glycaemic phenotype in pregnancy and rich omics data makes Gen3G similar in many ways to the Hyperglycaemia and Adverse Pregnancy Outcome follow-up study, with whom we have collaborated multiple times to replicate each other’s findings, adding to the robustness of our publications. Longitudinal cohort studies such as Gen3G are critical to elucidate the mechanisms supporting the DOHaD hypothesis in humans, which would help to develop novel prevention strategies. They also contribute to fostering collaborations between research teams and international consortia to create larger datasets to conduct meta-analyses and to validate results in diverse populations.

### Strengths and limitations

The Gen3G cohort has several strengths starting with its prospective design which allows collecting biological samples and anthropometric measures among other measures using standardised procedures, from the beginning of the pregnancy to 3 and 5 years after delivery. In addition, body composition was assessed in the majority of children during the 5-year follow-up visit, using DXA technology, which is considered the gold standard for body composition assessment.^68^ The large number of phenotypes we have in Gen3G allows considering many potential confounders in epidemiological analyses. Given the large number of omics data collected, future studies could apply artificial intelligence and deep learning algorithms to mine the relationships between biomarkers in pregnancy and their impacts on maternal and offspring health to advance precision medicine applications.^69^

We collected a variety of biological samples, including blood, hair (child only), stool and urine samples in both mothers and children, that will make it possible to explore new research hypotheses supported by mechanistic studies. We collected urine samples for assessment of albuminuria and podocyturia to monitor renal function and stool samples for assessment of gut microbiota, which is of interest since microbiota disruption has been associated with the development of T2D.^70^ In addition, hair samples will be used to assess cortisol levels in children, which have been linked to adiposity in childhood.^71^ We also performed a 75 g-OGTT in mothers at the 5-year follow-up visit to assess maternal glucose metabolism using dynamic measures.

Another strength of our study is the high retention rate of participants over the first 6–7 years of the study. Over 70% of the families who agreed to be contacted returned for the 3-year and 5-year follow-up visits. We used several strategies to optimise follow-up participation in the study, including stable research staff, sending annual Christmas cards to keep participants updated on the progress of the project and disseminating research findings in layman’s terms via social media. We updated participants’ contact details at each follow-up visit. Staff invited participants for follow-up visits during the school holidays (eg, summer vacations, spring breaks) and over the weekend days. We augmented research staff and trainees during the summer vacations to provide additional visits opportunities while children were not in school. Finally, for women who were unable to come to the research centre in person, we offered the opportunity to complete the questionnaires remotely, and for women who were able to come in person but had limited time, we offered shorter visits with fewer components (and the option of completing the questionnaires remotely).

The Gen3G cohort also has some limitations that are noteworthy to mention. First, our sample population is representative of the inhabitants of the Eastern Townships region and includes most individuals of French–Canadian ancestry, which limits the generalisability of our findings. However, Gen3G contributes to the Pregnancy And Childhood Epigenetics, EGG and GenDIP consortia which provide access to validate and replicate our results in other populations. Our sample size is also relatively small for some investigations (eg, genetic variants association studies), but reasonable for many omics studies, especially in the context of refined phenotyping and longitudinal follow-up of participants.

### Future plans

Gen3G recently engaged in its sixth follow-up, 10–12 years after birth, and we are inviting both the mother and the children to participate. In addition to conducting similar assessments as in the previous visits, including total fat mass and distribution quantification (DXA scans) and a 75 g-OGTT, we now assess detailed dietary intake and behaviours, including eating in the absence of hunger using standardised and validated functional tests,^72^ as well as standardised 24-hour recall.^73 74^ Furthermore, we measure hypothalamic volume, perfusion and connectivity with reward-system brain subregions using functional MRI in a subgroup of participants. The overarching goal of the sixth reassessment is to study how prenatal and early-life factors programme childhood adiposity and obesogenic dietary behaviours, and to investigate peripheral signals (eg, microbiota and hormones, including insulin and adipokines) contributing to central (hypothalamus) regulation of childhood adiposity and dietary behaviours. This follow-up should be completed by 2026. We used the Strengthening the Reporting of Observational Studies in Epidemiology cohort checklist when writing our report.^75^

### Collaboration

Collaboration propositions are welcome, either in the form of accessing questionnaire-based data and/or biological samples. Gen3G has a data and sample access policy. To discuss collaboration possibilities, investigators are invited to contact the corresponding author (LB) or M-FH and PP (mhivert@partners.org; patrice.perron@usherbrooke.ca). Furthermore, we deposited many omics and phenotype data in Gene Expression Omnibus (GEO) (GSE216997) and the database of Genotypes and Phenotypes (dbGap) (accession # phs003151.v1.p1).

## supplementary material

10.1136/bmjopen-2024-093434 online supplemental file 1

10.1136/bmjopen-2024-093434 online supplemental file 2

## Data Availability

Data are available in a public, open access repository.
