# Supplementary material for: Cohort profile: the Genetics of Glucose regulation in Gestation and Growth (Gen3G) – a prospective prebirth cohort of mother–child pairs in Sherbrooke, Canada, 3-year and 5-year follow-up visits
Source: BMJ Open. 2025 Mar 22;15(3):e093434. doi: 10.1136/bmjopen-2024-093434 (PMC11931902; doi:10.1136/bmjopen-2024-093434)
Supplement: online supplemental file 1 [file bmjopen-15-3-s001.pdf]

	Mean of %CV intraassay	Mean of %CV interassay
C-peptid	0.91	17.73
Insulin	0.89	28.62
Leptin	1.33	22.72
MCP-1	1.37	12.49
TNF-α	1.63	23.33
Adiponectin*	0.95	46.89
PAI-1*	1.04	31.12

*For our 3 internal controls, the concentrations obtained were outside the range of linearity of the standard curves, hence the %CV higher than recommended by the manufacturer.

For each analyte, we calculated the mean of intraassay %CV of all assays. For interassay %CV, we used 3 internal controls that were repeated on every assay. Overall mean of %CV was obtained by calculating the mean of the means of the 3 internal controls.
