# Supplementary material for: Cohort profile: the Genetics of Glucose regulation in Gestation and Growth (Gen3G) – a prospective prebirth cohort of mother–child pairs in Sherbrooke, Canada, 3-year and 5-year follow-up visits
Source: BMJ Open. 2025 Mar 22;15(3):e093434. doi: 10.1136/bmjopen-2024-093434 (PMC11931902; doi:10.1136/bmjopen-2024-093434)
Supplement: online supplemental file 2 [file bmjopen-15-3-s002.pdf]

Supplementary Table 2. List of references published with the Gen3G cohort since its inception.

| | References |
|---|---|
| 1 | Lacroix M, Battista MC, Doyon M, Ménard J, Ardilouze JL, Perron P, Hivert MF. Lower adiponectin levels at first trimester of pregnancy are associated with increased insulin resistance and higher risk of developing gestational diabetes mellitus. Diabetes Care. 2013 Jun;36(6):1577-83. doi: 10.2337/dc12-1731. |
| 2 | Guillemette L, Lacroix M, Battista MC, Doyon M, Moreau J, Ménard J, Ardilouze JL, Perron P, Hivert MF. TNFα dynamics during the oral glucose tolerance test vary according to the level of insulin resistance in pregnant women. J Clin Endocrinol Metab. 2014 May;99(5):1862-9. doi: 10.1210/jc.2013-4016. |
| 3 | Lacroix M, Battista MC, Doyon M, Houde G, Ménard J, Ardilouze JL, Hivert MF, Perron P. Lower vitamin D levels at first trimester are associated with higher risk of developing gestational diabetes mellitus. Acta Diabetol. 2014 Aug;51(4):609-16. doi: 10.1007/s00592-014-0564-4. |
| 4 | Allard C, Desgagné V, Patenaude J, Lacroix M, Guillemette L, Battista MC, Doyon M, Ménard J, Ardilouze JL, Perron P, Bouchard L, Hivert MF. Mendelian randomization supports causality between maternal hyperglycemia and epigenetic regulation of leptin gene in newborns. Epigenetics. 2015 Apr; 10(4): 342–351. doi: 10.1080/15592294.2015.1029700. |
| 5 | Guillemette L, Lacroix M, Allard C, Patenaude J, Battista MC, Doyon M, Moreau J, Ménard J, Ardilouze JL, Perron P, Côté AM, Hivert MF. Preeclampsia is associated with an increased pro-inflammatory profile in newborns. J Reprod Immunol. 2015 Nov;112:111-4. doi: 10.1016/j.jri.2015.09.003. |
| 6 | Guillemette L, Allard C, Lacroix M, Patenaude J, Battista MC, Doyon M, Moreau J, Ménard J, Bouchard L, Ardilouze JL, Perron P, Hivert MF. Genetics of Glucose regulation in Gestation and Growth (Gen3G): a prospective prebirth cohort of mother–child pairs in Sherbrooke, Canada. BMJ Open. 2016; 6(2): e010031. doi: 10.1136/bmjopen-2015-010031. |
| 7 | Ruchat SM, Allard C, Doyon M, Lacroix M, Guillemette L, Patenaude J, Battista MC, Ardilouze JL, Perron P, Bouchard L, Hivert MF. Timing of Excessive Weight Gain During Pregnancy Modulates Newborn Anthropometry. J Obstet Gynaecol Can. 2016 Feb;38(2):108-17. doi: 10.1016/j.jogc.2015.12.014. |
| 8 | Lacroix M, Battista MC, Doyon, Moreau J, Patenaude J, Guillemette L, Ménard J, Ardilouze JL, Perron P, Hivert MF. Higher maternal leptin levels at second trimester are associated with subsequent greater gestational weight gain in late pregnancy. BMC Pregnancy Childbirth. 2016; 16: 62. doi: 10.1186/s12884-016-0842-y. |
| 9 | Tyrrell J, Richmond RC, Palmer TM, Feenstra B, Rangarajan J, Metrustry S, Cavadino A, Paternoster L, Armstrong LL, De Silva NM, Wood AR, Horikoshi M, Geller F, Myhre R, et al. Early Growth Genetics (EGG) Consortium. Genetic Evidence for Causal Relationships Between Maternal Obesity-Related Traits and Birth Weight. JAMA. 2016 Mar 15;315(11):1129-40. doi: 10.1001/jama.2016.1975. Erratum in: JAMA. 2016 Apr 19;315(15):1661. doi: 10.1001/jama.2016.3680. |

| | |
|---|---|
| 10 | Powe CE, Allard C, Battista MC, Doyon M, Bouchard L, Ecker JL, Perron P, Florez JC, Thadhani R, Hivert MF. Heterogeneous Contribution of Insulin Sensitivity and Secretion Defects to Gestational Diabetes Mellitus. Diabetes Care. 2016 Jun;39(6):1052-5. doi: 10.2337/dc15-2672. |

| | |
|---|---|
| 11 | Côté S, Gagné-Ouellet V, Guay SP, Allard C, Houde AA, Perron P, Baillargeon JP, Gaudet D, Guérin R, Brisson D, Hivert MF, Bouchard L. PPARGC1α gene DNA methylation variations in human placenta mediate the link between maternal hyperglycemia and leptin levels in newborns. Clin Epigenetics. 2016 Jun 22;8:72. doi: 10.1186/s13148-016-0239-9. |
| 12 | Cardenas A, Allard C, Doyon M, Houseman EA, Bakulski KM, Perron P, Bouchard L, Hivert MF. Validation of a DNA methylation reference panel for the estimation of nucleated cells types in cord blood. Epigenetics. 2016 Nov;11(11):773-779. doi: 10.1080/15592294.2016.1233091. |
| 13 | Patenaude J, Lacerte G, Lacroix M, Guillemette L, Allard C, Doyon M, Battista MC, Moreau J, Ménard J, Ardilouze JL, Perron P, Hivert MF. Associations of Maternal Leptin with Neonatal Adiposity Differ according to Pregravid Weight. Neonatology. 2017;111(4):344-352. doi: 10.1159/000454756. |
| 14 | Hivert MF, Scholtens DM, Allard C, Nodzenski M, Bouchard L, Brisson D, Lowe LP, McDowell I, Reddy T, Dastani Z, Richards JB, Hayes MG, Lowe WL Jr. Genetic determinants of adiponectin regulation revealed by pregnancy. Obesity (Silver Spring). 2017 May;25(5):935-944. doi: 10.1002/oby.21805. |
| 15 | Gagné-Ouellet V, Houde AA, Guay SP, Perron P, Gaudet D, Guérin R, Baillargeon JP, Hivert MF, Brisson D, Bouchard L. Placental lipoprotein lipase DNA methylation alterations are associated with gestational diabetes and body composition at 5 years of age. Epigenetics. 2017 Aug;12(8):616-625. doi: 10.1080/15592294.2017.1322254. |
| 16 | Sharp GC, Salas LA, Monnereau C, Allard C, Yousefi P, and al. Maternal BMI at the start of pregnancy and offspring epigenome-wide DNA methylation: findings from the pregnancy and childhood epigenetics (PACE) consortium. Hum Mol Genet. 2017 Oct 15; 26(20): 4067–4085. doi: 10.1093/hmg/ddx290. |
| 17 | Felix JF, Joubert BR, Baccarelli AA, Sharp GC, Almqvist C, and al. Cohort Profile: Pregnancy And Childhood Epigenetics (PACE) Consortium. Int J Epidemiol. 2018 Feb; 47(1): 22–23u. doi: 10.1093/ije/dyx190. Correction in: Int J Epidemiol. 2018 Feb; 47(1): 24. |
| 18 | Switkowski KM, Camargo CA, Perron P, Rifas-Shiman SL, Oken E, Hivert MF. Cord Blood Vitamin D Status Is Associated With Cord Blood Insulin and C-Peptide in Two Cohorts of Mother-Newborn Pairs. J Clin Endocrinol Metab. 2019 Sep 1;104(9):37853794. doi: 10.1210/jc.2018-02550. |
| 19 | Powe CE, Nodzenski M, Talbot O, Allard C, Briggs C, Leya MV, Perron P, Bouchard L, Florez JC, Scholtens DM, Lowe WL Jr, Hivert MF. Genetic Determinants of Glycemic Traits and the Risk of Gestational Diabetes Mellitus. Diabetes. 2018 Dec;67(12):2703-2709. doi: 10.2337/db18-0203. |

| | |
|---|---|
| 20 | Santos S, Eekhout I, Voerman E, Gaillard R, Barros H, Charles MA, and al. Gestational weight gain charts for different body mass index groups for women in Europe, North America, and Oceania. BMC Med. 2018; 16: 201. doi: 10.1186/s12916-018-1189-1. |
| 21 | Blais S, Patenaude J, Doyon M, Bouchard L, Perron P, Hivert MF, Dallaire F. Effect of gestational diabetes and insulin resistance on offspring's myocardial relaxation kinetics at three years of age. PLoS One. 2018 Nov 21;13(11):e0207632. doi: 10.1371/journal.pone.0207632. |

| | |
|---|---|
| 22 | Lesseur C, Chen J. Adverse Maternal Metabolic Intrauterine Environment and Placental Epigenetics: Implications for Fetal Metabolic Programming. Curr Environ Health Rep. 2018 Dec;5(4):531-543. doi: 10.1007/s40572-018-0217-9. |
| 23 | Layton J, Powe C, Allard C, Battista MC, Doyon M, Bouchard L, Perron P, Wessel J, Hivert MF. Maternal lipid profile differs by gestational diabetes physiologic subtype. Metabolism. 2019 Feb;91:39-42. doi: 10.1016/j.metabol.2018.11.008. |
| 24 | Middeldorp CM, Felix JF, Mahajan A; EArly Genetics Lifecourse Epidemiology (EAGLE) consortium; Early Growth Genetics (EGG) consortium; McCarthy MI. The Early Growth Genetics (EGG) and EArly Genetics and Lifecourse Epidemiology (EAGLE) consortia: design, results and future prospects. Eur J Epidemiol. 2019 Mar;34(3):279-300. doi: 10.1007/s10654-019-00502-9. |
| 25 | Ma B, Allard C, Bouchard L, Perron P, Mittleman MA, Hivert MF, Liang L. Locusspecific DNA methylation prediction in cord blood and placenta. Epigenetics. 2019 Apr;14(4):405-420. doi: 10.1080/15592294.2019.1588685. |
| 26 | Franzago M, Fraticelli F, Stuppia L, Vitacolonna E. Nutrigenetics, epigenetics and gestational diabetes: consequences in mother and child. Epigenetics. 2019 Mar;14(3):215-235. doi: 10.1080/15592294.2019.1582277. |
| 27 | Küpers LK, Monnereau C, Sharp GC, Yousefi P, Salas LA and al. Meta-analysis of epigenome-wide association studies in neonates reveals widespread differential DNA methylation associated with birthweight. Nat Commun. 2019; 10: 1893. doi: 10.1038/s41467-019-09671-3. |
| 28 | LifeCycle Project-Maternal Obesity and Childhood Outcomes Study Group; Voerman E, Santos S, Inskip H, Amiano P, Barros H, Charles MA, Chatzi L, Chrousos GP, and al. Association of Gestational Weight Gain With Adverse Maternal and Infant Outcomes.<br>JAMA. 2019 May 7;321(17):1702-1715. doi: 10.1001/jama.2019.3820. |
| 29 | Switkowski KM, Camargo CA, Perron P, Rifas-Shiman SL, Oken E, Hivert MF. Cord Blood Vitamin D Status Is Associated With Cord Blood Insulin and C-Peptide in Two Cohorts of Mother-Newborn Pairs. J Clin Endocrinol Metab. 2019 Sep 1;104(9):37853794. doi: 10.1210/jc.2018-02550. |
| 30 | Fernandez-Jimenez N, Allard C, Bouchard L, Perron P, Bustamante M, Bilbao JR, Hivert MF. Comparison of Illumina 450K and EPIC arrays in placental DNA methylation. Epigenetics. 2019 Dec;14(12):1177-1182. doi: 10.1080/15592294.2019.1634975. |

| | |
|---|---|
| 31 | Cardenas A, Lutz SM, Everson TM, Perron P, Bouchard L, Hivert MF. Mediation by Placental DNA Methylation of the Association of Prenatal Maternal Smoking and Birth Weight. Am J Epidemiol. 2019 Nov; 188(11): 1878–1886. doi: 10.1093/aje/kwz184. Correction in: Am J Epidemiol. 2020 Oct; 189(10): 1212. |
| 32 | Breton E, Gagné-Ouellet V, Thibeault K, Guérin R, Van Lieshout R, Perron P, Hivert M, Bouchard L. Placental NEGR1 DNA methylation is associated with BMI and neurodevelopment in preschool-age children. Epigenetics. 2020; 15(3): 323–335. doi: 10.1080/15592294.2019.1666653. |
| 33 | Gagné-Ouellet V, Breton E, Thibeault K, Fortin CA, Cardenas A, Guérin R, Perron P, Hivert MF, Bouchard L. Mediation Analysis Supports a Causal Relationship between Maternal Hyperglycemia and Placental DNA Methylation Variations at the Leptin Gene Locus and Cord Blood Leptin Levels. Int J Mol Sci. 2020 Jan; 21(1): 329. doi: 10.3390/ijms21010329. |

| | |
|---|---|
| 34 | Doyon M, Pelland-St-Pierre L, Allard C, Bouchard L, Perron P, Hivert MF. Associations of sleep duration, sedentary behaviours and energy expenditure with maternal glycemia in pregnancy. Sleep Med. 2020 Jan;65:54-61. doi: 10.1016/j.sleep.2019.07.008. |
| 35 | Merid SK, Novoloaca A, Sharp GC, Küpers LK, Kho AT, Roy R, Gao L, AnnesiMaesano I, Jain P, Plusquin M, and al. Epigenome-wide meta-analysis of blood DNA methylation in newborns and children identifies numerous loci related to gestational age.<br>Genome Med. 2020; 12: 25.. doi: 10.1186/s13073-020-0716-9. |
| 36 | Differding M, Hivert MF, Doyon M, Bouchard L, Perron P, Guérin R, Massé É, Mueller<br>N. Gut Microbiome Composition Is Associated with Blood Pressure in Mother-Child Pairs 5 Years After Birth. Curr Dev Nutr. 2020 Jun; 4(Suppl 2): 1555.<br>doi: 10.1093/cdn/nzaa062_012 |
| 37 | Differding MK, Doyon M, Bouchard L, Perron P, Guérin R, Asselin C, Massé E, Hivert MF, Mueller NT. Potential interaction between timing of infant complementary feeding and breastfeeding duration in determination of early childhood gut microbiota composition and BMI. Pediatr Obes. 2020 Aug; 15(8): e12642. doi: 10.1111/ijpo.12642. |
| 38 | Gagné-Ouellet V, Breton E, Thibeault K, Fortin CA, Desgagné V, Girard Tremblay É, Cardenas A, Guérin R, Perron P, Hivert MF, Bouchard L. Placental Epigenome-Wide Association Study Identified Loci Associated with Childhood Adiposity at 3 Years of Age. Int J Mol Sci. 2020 Oct; 21(19): 7201. doi: 10.3390/ijms21197201. |
| 39 | Powe CE, Hivert MF, Udler MS. Defining Heterogeneity Among Women With Gestational Diabetes Mellitus. Diabetes. 2020 Oct; 69(10): 2064–2074. doi: 10.2337/dbi20-0004. |
| 40 | Hivert MF, Cardenas A, Allard C, Doyon M, Powe CE, Catalano PM, Perron P, Bouchard L. Interplay of Placental DNA Methylation and Maternal Insulin Sensitivity in Pregnancy. Diabetes. 2020 Mar; 69(3): 484–492. doi: 10.2337/db19-0798. |

| | |
|---|---|
| 41 | Powe CE, Udler MS, Hsu S, Allard C, Kuang A, Manning AK, Perron P, Bouchard L, Lowe WL Jr, Scholtens D, Florez JC, Hivert MF. Genetic Loci and Physiologic Pathways Involved in Gestational Diabetes Mellitus Implicated Through Clustering. Diabetes. 2021 Jan; 70(1): 268–281. doi: 10.2337/db20-0772. |
| 42 | Juvinao-Quintero DL, Starling AP, Cardenas A, Powe CE, Perron P, Bouchard L, Dabelea D, Hivert MF. Epigenome-wide association study of maternal hemoglobin A1c in pregnancy and cord blood DNA methylation. Epigenomics. 2021 Feb; 13(3): 203–218. doi: 10.2217/epi-2020-0279. |
| 43 | Lent S, Cardenas A, Rifas-Shiman SL, Perron P, Bouchard L, Liu CT, Hivert MF, Dupuis J. Detecting differentially methylated regions with multiple distinct associations. Epigenomics. 2021 Mar; 13(6): 451–464. doi: 10.2217/epi-2020-0344. |
| 44 | Rahman ML, Doyon M, Arguin M, Perron P, Bouchard L, Hivert MF. A prospective study of maternal adiposity and glycemic traits across pregnancy and mid-childhood metabolomic profiles. Int J Obes (Lond). 2021 Apr;45(4):860-869. doi: 10.1038/s41366021-00750-4. |
| 45 | Blais K, Arguin M, Allard C, Doyon M, Dolinsky VW, Bouchard L, Hivert MF, Perron P. Maternal glucose in pregnancy is associated with child's adiposity and leptin at 5 years of age. Pediatr Obes. 2021 Sep;16(9):e12788. doi: 10.1111/ijpo.12788. |
| 46 | Lu T, Cardenas A, Perron P, Hivert MF, Bouchard L, Greenwood CMT. Greenwood. Detecting cord blood cell type-specific epigenetic associations with gestational diabetes mellitus and early childhood growth. Clin Epigenetics. 2021; 13: 131. doi: 10.1186/s13148-021-01114-5. |
| 47 | Juvinao-Quintero DL, Cardenas A, Perron P, Bouchard L, Lutz SM, Hivert MF. Associations between an integrated component of maternal glycemic regulation in pregnancy and cord blood DNA methylation. Epigenomics. 2021 Sep; 13(18): 1459–1472. doi: 10.2217/epi-2021-0220. |
| 48 | Faleschini S, Doyon M, Arguin M, Perron P, Bouchard L, Hivert MF. Associations of maternal insulin resistance during pregnancy and offspring inflammation at birth and at 5 years of age: A prospective study in the Gen3G cohort. Cytokine. 2021 Oct; 146:155636. doi: 10.1016/j.cyto.2021.155636. |
| 49 | Alvarado-Flores F, Kaneko-Tarui T, Beyer W, Katz J, Chu T, Catalano P, Sadovsky Y, Hivert MF, O'Tierney-Ginn P. Placental miR-3940-3p Is Associated With Maternal Insulin Resistance in Late Pregnancy. J Clin Endocrinol Metab. 2021 Dec; 106(12): 3526–3535. doi: 10.1210/clinem/dgab571. |
| 50 | Everson TM, Vives-Usano M, Seyve E, Cardenas A, Lacasaña M, Craig JM, Lesseur C, Baker ER, Fernandez-Jimenez N, and al. Placental DNA methylation signatures of maternal smoking during pregnancy and potential impacts on fetal growth. Nat Commun. 2021; 12: 5095. doi: 10.1038/s41467-021-24558-y. |

| | |
|---|---|
| 51 | Briollais L, Rustand D, Allard C, Wu Y, Xu J, Rajan SG, Hivert MF, Doyon M, Bouchard L, McGowan PO, Matthews S, Lye S. DNA methylation mediates the association between breastfeeding and early-life growth trajectories. Clin Epigenetics. 2021; 13: 231. doi: 10.1186/s13148-021-01209-z. |
| 52 | Shorey-Kendrick LE, McEvoy CT, O'Sullivan SM, Milner K, Vuylsteke B, Tepper RS, Haas DM, Park B, Gao L, Vu A, Morris CD, Spindel ER. Impact of vitamin C supplementation on placental DNA methylation changes related to maternal smoking: association with gene expression and respiratory outcomes. Clin Epigenetics. 2021; 13: 177. doi: 10.1186/s13148-021-01161-y. |
| 53 | Ghildayal N, Fore R, Lutz SM, Cardenas A, Perron P, Bouchard L, Hivert MF. Earlypregnancy maternal body mass index is associated with common DNA methylation markers in cord blood and placenta: a paired-tissue epigenome-wide association study. Epigenetics. 2022; 17(7): 808–818. doi: 10.1080/15592294.2021.1959975. |
| 54 | Fujii R, Sato S, Tsuboi Y, Cardenas A, Suzuki K. DNA methylation as a mediator of associations between the environment and chronic diseases: A scoping review on application of mediation analysis. Epigenetics. 2022; 17(7): 759–785. doi: 10.1080/15592294.2021.1959736. |
| 55 | Tobi EW, Juvinao-Quintero DL, Ronkainen J, Ott R, Alfano R, Canouil M, Geurtsen ML, Khamis A, Küpers LK, and al. Maternal Glycemic Dysregulation During Pregnancy and Neonatal Blood DNA Methylation: Meta-analyses of Epigenome-Wide Association Studies. Diabetes Care. 2022 Mar; 45(3): 614–623. Mar 4. doi: 10.2337/dc21-1701. |
| 56 | Alenezi WM, Milano L, Fierheller CT, Serruya C, Revil T, Oros KK, Behl S, Arcand SL, Nayar P, and al. The Genetic and Molecular Analyses of RAD51C and RAD51D Identifies Rare Variants Implicated in Hereditary Ovarian Cancer from a Genetically Unique Population. Cancers (Basel) 2022 May; 14(9): 2251. doi: 10.3390/cancers14092251 |
| 57 | Thibeault K, Légaré C, Desgagné V, White F, Clément AA, Scott MS, Jacques PÉ, Guérin R, Perron P, Hivert MF, Bouchard L. Maternal Body Mass Index Is Associated with Profile Variation in Circulating MicroRNAs at First Trimester of Pregnancy. Biomedicines. 2022 Jul; 10(7): 1726. doi: 10.3390/biomedicines10071726. |
| 58 | Thibeault K, Légaré C, Desgagné V, White F, Clément AA, Scott MS, Jacques PÉ, Guérin R, Perron P, Hivert MF, Bouchard L. Maternal Body Mass Index Is Associated with Profile Variation in Circulating MicroRNAs at First Trimester of Pregnancy. Biomedicines. 2022 Jul; 10(7): 1726. doi: 10.3390/biomedicines10071726. |
| 60 | Légaré C, Clément AA, Desgagné V, Thibeault K, White F, Guay SP, Arsenault BJ, Scott MS, Jacques PÉ, Perron P, Guérin R, Hivert MF, Bouchard L. Human plasma pregnancy-associated miRNAs and their temporal variation within the first trimester of pregnancy. Reprod Biol Endocrinol. 2022; 20: 14. doi: 10.1186/s12958-021-00883-1. |
| 61 | Pervjakova N, Moen GH, Borges MC, Ferreira T, Cook JP, Allard C, Beaumont RN, Canouil M, Hatem G, and al. Multi-ancestry genome-wide association study of gestational diabetes mellitus highlights genetic links with type 2 diabetes. Hum Mol Genet. 2022 Oct 1; 31(19): 3377–3391. doi: 10.1093/hmg/ddac050. |

| | |
|---|---|
| 62 | Taschereau A, Desgagné V, Faleschini S, Guérin R, Allard C, Perron P, Hivert MF, Bouchard L. SERPINE1 DNA Methylation Levels Quantified in Blood Cells at Five Years of Age Are Associated with Adiposity and Plasma PAI-1 Levels at Five Years of Age. Int J Mol Sci. 2022 Oct; 23(19): 11833. doi: 10.3390/ijms231911833. |
| 63 | Légaré C, Desgagné V, Poirier C, Thibeault K, White F, Clément AA, Scott MS, Jacques PÉ, Perron P, Guérin R, Hivert MF, Bouchard L. First trimester plasma microRNAs levels predict Matsuda Index-estimated insulin sensitivity between 24th and 29th week of pregnancy. BMJ Open Diabetes Res Care. 2022; 10(2): e002703. doi: 10.1136/bmjdrc-2021-002703. |
| 64 | Solomon O, Huen K, Yousefi P, Küpers LK, González JR, Suderman M, Reese SE, Page CM, Gruzieva O, and al. Meta-analysis of epigenome-wide association studies in newborns and children show widespread sex differences in blood DNA methylation. Mutat Res Rev Mutat Res. 2022 Jan-Jun; 789: 108415. doi: 10.1016/j.mrrev.2022.108415. |
| 65 | Lapehn S, Paquette AG. The Placental Epigenome as a Molecular Link Between Prenatal Exposures and Fetal Health Outcomes Through the DOHaD Hypothesis. Curr Environ Health Rep. 2022; 9(3): 490–501. doi: 10.1007/s40572-022-00354-8. |
| 66 | Légaré C, Desgagné V, Thibeault K, White F, Clément AA, Poirier C, Luo ZC, Scott MS, Jacques PÉ, Perron P, Guérin R, Hivert MF, Bouchard L. First Trimester Plasma MicroRNA Levels Predict Risk of Developing Gestational Diabetes Mellitus. Front Endocrinol (Lausanne) 2022; 13: 928508. doi: 10.3389/fendo.2022.928508. |
| 67 | Fernandez-Jimenez N, Fore R, Cilleros-Portet A, Lepeule J, Perron P, Kvist T, Tian FY, Lesseur C, Binder AM, and al. A meta-analysis of pre-pregnancy maternal body mass index and placental DNA methylation identifies 27 CpG sites with implications for mother-child health. Commun Biol. 2022; 5: 1313. doi: 10.1038/s42003-022-04267-y. |
| 68 | Wu FY, Yin RX. Recent progress in epigenetics of obesity. Diabetol Metab Syndr. 2022; 14: 171. doi: 10.1186/s13098-022-00947-1. |
| 69 | Jääskeläinen T, Klemetti MM. Genetic Risk Factors and Gene–Lifestyle Interactions in Gestational Diabetes. Nutrients. 2022 Nov; 14(22): 4799. doi: 10.3390/nu14224799. |
| 70 | Blais K, Doyon M, Arguin M, Bouchard L, Perron P, Hivert MF. Associations between Cord Blood Leptin Levels and Childhood Adiposity Differ by Sex and Age at Adiposity Assessment. Life (Basel) 2022 Dec; 12(12): 2060. doi: 10.3390/life12122060. |

| | |
|---|---|
| 71 | Cohen N, Faleschini S, Rifas-Shiman SL, Bouchard L, Doyon M, Simard O, Arguin M, Fink G, Alman AC, Kirby R, Chen H, Wilson R, Fryer K, Perron P, Oken E, Hivert MF. Associations of maternal glucose markers in pregnancy with cord blood glucocorticoids and child hair cortisol levels. J Dev Orig Health Dis. 2023 Feb; 14(1): 88–95. doi: 10.1017/S2040174422000381. |
| 72 | Ghildayal N, Allard C, Blais K, Doyon M, Arguin M, Bouchard L, Perron P, Hivert MF. Associations of maternal insulin sensitivity during pregnancy with childhood central adiposity in the Genetics of Glucose regulation in Gestation and Growth cohort. Pediatr Obes. 2023 Feb; 18(2): e12982. doi: 10.1111/ijpo.12982. |

| | |
|---|---|
| 73 | Caitlin T. Fierheller, Wejdan M. Alenezi, Corinne Serruya, Timothée Revil, Setor Amuzu, Karine Bedard, Deepak N. Subramanian, Eleanor Fewings, and al. Molecular Genetic Characteristics of FANCI, a Proposed New Ovarian Cancer Predisposing Gene. Genes (Basel) 2023 Feb; 14(2): 277. doi: 10.3390/genes14020277. |
| 74 | Wejdan M. Alenezi, Caitlin T. Fierheller, Corinne Serruya, Timothée Revil, Kathleen K. Oros, Deepak N. Subramanian, Jeffrey Bruce, Dan Spiegelman, Trevor Pugh, and al. Genetic analyses of DNA repair pathway associated genes implicate new candidate cancer predisposing genes in ancestrally defined ovarian cancer cases.Front Oncol. 2023; 13: 1111191. Published online 2023 Mar 8. doi: 10.3389/fonc.2023.1111191. |
| 75 | Mortillo M, Marsit CJ. Select early-life environmental exposures and DNA methylation in the placenta. Curr Environ Health Rep. 2023 Mar; 10(1): 22–34. doi: 10.1007/s40572022-00385-1. |
| 76 | Liu T, Jia F, Differding MK, Zhao N, Doyon M, Bouchard L, Perron P, Guérin R, Massé E, Hivert MF, Mueller NT. Pre-pregnancy body mass index and gut microbiota of mothers and children 5 years postpartum. Int J Obes (Lond). 2023 Sep; 47(9): 807–816. doi: 10.1038/s41366-023-01322-4. |
| 77 | Taschereau A, Thibeault K, Allard C, Juvinao-Quintero D, Perron P, Lutz SM, Bouchard L, Hivert MF. Maternal glycemia in pregnancy is longitudinally associated with blood DNAm variation at the FSD1L gene from birth to 5 years of age. Clin Epigenetics. 2023; 15: 107. doi: 10.1186/s13148-023-01524-7. |
| 78 | Kadalayil L, Alam MZ, White CH, Ghantous A, Walton E, Gruzieva O, Merid SK, Kumar A, Roy RP, and al. Analysis of DNA methylation at birth and in childhood reveals changes associated with season of birth and latitude. Clin Epigenetics. 2023; 15: 148. doi: 10.1186/s13148-023-01542-5. |
| 79 | Faleschini S, Doyon M, Arguin M, Lepage JF, Tiemeier H, Van Lieshout RJ, Perron P, Bouchard L, Hivert MF. Maternal Hyperglycemia in Pregnancy and Offspring Internalizing and Externalizing Behaviors. Matern Child Health J. 2023 Oct;27(10):1765-1773. doi: 10.1007/s10995-023-03706-4. |
| 80 | Beaumont RN, Flatley C, Vaudel M, Wu X, Chen J, Moen GH, Skotte L, Helgeland Ø, Solé-Navais P, Banasik K, and al. Genome-wide association study of placental weight identifies distinct and shared genetic influences between placental and fetal growth. Nat Genet. 2023; 55(11): 1807–1819. doi: 10.1038/s41588-023-01520-w. |
| 81 | Hivert MF, White F, Allard C, James K, Majid S, Aguet F, Ardlie K, Edlow A, Florez J, Bouchard L, Jacques PE, Karumanchi S, Powe C. Placental RNA sequencing implicates IGFBP1 in insulin sensitivity during pregnancy and in gestational diabetes. Version 1. Res Sq. Preprint. 2023 Oct 27. doi: 10.21203/rs.3.rs-3464151/v1 Published in: Nat Med. 2024; 30(6): 1689–1695. |
| 82 | Borges MC, Clayton GL, Freathy RM, Felix JF, Fernández-Sanlés A, Soares AG, Kilpi F, Yang Q, and al. Integrating multiple lines of evidence to assess the effects of maternal BMI on pregnancy and perinatal outcomes. BMC Med. 2024; 22: 32. doi: 10.1186/s12916-023-03167-0. |

| | |
|---|---|
| 83 | Sordillo JE, White F, Majid S, Aguet F, Ardlie KG, Karumanchi SA, Florez JC, Powe CE, Edlow AG, Bouchard L, Jacques PE, Hivert MF. Higher Maternal Body Mass Index Is Associated With Lower Placental Expression of EPYC: A Genome-Wide Transcriptomic Study. J Clin Endocrinol Metab. 2024 Mar; 109(3): e1159–e1166. doi: 10.1210/clinem/dgad619. |
| 84 | Hivert MF, White F, Allard C, James K, Majid S, Aguet F, Ardlie KG, Florez JC, Edlow AG, Bouchard L, Jacques PÉ, Karumanchi SA, Powe CE. Placental IGFBP1 levels during early pregnancy and the risk of insulin resistance and gestational diabetes. Nat Med. 2024; 30(6): 1689–1695. doi: 10.1038/s41591-024-02936-5. |
| 85 | Yeung E, Biedrzycki RJ, Gómez Herrera LC, Issarapu P, Dou J, Marques IF, Mansuri SR, Page CM, and al. Maternal age is related to offspring DNA methylation: A metaanalysis of results from the PACE consortium. Aging Cell. 2024 Aug; 23(8): e14194. doi: 10.1111/acel.14194. |
| 86 | Légaré C, Desgagné V, Thibeault K, White F, Clément AA, Poirier C, Luo ZC, Scott MS, Jacques PÉ, Perron P, Guérin R, Hivert MF, Bouchard L. First-Trimester Plasmatic microRNAs Are Associated with Fasting Glucose Levels in Late Second Trimester of Pregnancy. Biomedicines. 2024 Jun; 12(6): 1285. doi: 10.3390/biomedicines12061285. |
| 87 | Gunter-Rahman F, Mallett S, White F, Jacques PÉ, Raju RM, Hivert MF, Lee EA. Maternal obesity and offspring neurodevelopment are associated with hypoxic gene expression in term human placenta. Version 1. bioRxiv. Preprint. 2024 Jul 10. doi: 10.1101/2024.07.10.602900. |
| 88 | Bohn B, Tilves C, Chen Y, Doyon M, Bouchard L, Perron P, Guérin R, Massé É, Hivert MF, Mueller NT. Associations of gut microbiota features and circulating metabolites with systemic inflammation in children. BMJ Open Gastroenterol. 2024; 11(1): e001470. doi: 10.1136/bmjgast-2024-001470. |
| 89 | Semnani-Azad Z, Rahman ML, Arguin M, Doyon M, Perron P, Bouchard L, Hivert MF. Plasma metabolomic profile of adiposity and body composition in childhood: The Genetics of Glucose regulation in Gestation and Growth cohort. Pediatr Obes. 2024 Sep;19(9):e13149. doi: 10.1111/ijpo.13149. |
| 90 | Marchildon C, Arguin M, Doyon M, Cote S, Michaud A, Huck J, Gingras V, Perron P, Hivert MF, Bouchard L, Whittingstall K. Maternal Blood Glucose And Hypothalamus Volume: Novel Insights For Intergenerational Transmission Of Endocrine Traits. J Endocr Soc. 2024 Oct 5; 8(Suppl 1): bvae163.839. doi: 10.1210/jendso/bvae163.839 |
| 91 | Diemer EW, Tuhkanen J, Sammallahti S, Heinonen K, Neumann A, Robinson SL, Suderman M, Jin J, Page CM, Fore R, Rifas-Shiman SL, Oken E, Perron P, Bouchard L, Hivert MF, Räikköne K, Lahti J, Yeung EH, Guan W, Mumford SL, Magnus MC, Håberg S, Nystad W, Parr CL, London SJ, Felix JF, Tiemeier H. Epigenome-wide metaanalysis of prenatal vitamin D insufficiency and cord blood DNA methylation Epigenetics. 2024; 19(1): 2413815. doi: 10.1080/15592294.2024.2413815. |
