# Supplementary material for: Cohort profile: the Genetics of Glucose regulation in Gestation and Growth (Gen3G) – a prospective prebirth cohort of mother–child pairs in Sherbrooke, Canada, 3-year and 5-year follow-up visits
Source: BMJ Open. 2025 Mar 22;15(3):e093434. doi: 10.1136/bmjopen-2024-093434 (PMC11931902; doi:10.1136/bmjopen-2024-093434)
Supplement: Supplementary file 3 [file bmjopen-2024-093434.reviewer_comments.pdf]

PEER REVIEW HISTORY

BMJ Open publishes all reviews undertaken for accepted manuscripts. Reviewers are asked to complete a checklist review form and are provided with free text boxes to elaborate on their assessment. These free text comments are reproduced below.

ARTICLE DETAILS

Title (Provisional)

Cohort profile: The Genetics of Glucose regulation in Gestation and Growth (Gen3G) - a prospective prebirth cohort of mother–child pairs in Sherbrooke, Canada, 3- and 5-years follow-up visits.

Authors

Taschereau, Amelie; Doyon, Myriam; Arguin, Mélina; Allard, Catherine; Desgagné, Véronique; Cote, Anne-Marie; Massé, Éric; Jacques, Pierre-Étienne; Perron, Patrice; Hivert, Marie-France; Bouchard, Luigi

---

VERSION 1 - REVIEW

**Reviewer**	**1**
**Name**	**Foley, Helen**
**Affiliation**	**USC Keck School of Medicine**
**Date**	**06-Nov-2024**
**COI**	**None**

Title: Cohort profile: The Genetics of Glucose regulation in Gestation and Growth (Gen3G) - a prospective prebirth cohort of mother–child pairs in Sherbrooke, Canada, 3- and 5-year post-partum follow-ups.

Recommending: Minor Revisions

Summary:
This cohort update to the Gen3G study includes important developments in child health outcomes at 3 and 5 years post-birth. The cohort has followed up on a substantial number of their participants, and collected a biobank of a variety of samples as well as useful assay and omics data. While the sample is limited to those of French-Canadian ancestry, the cohort has contributed to several consortia to better understand glucose dysregulation. This manuscript describes the contributions and findings to date of the Gen3G cohort and its plans for the 6th followup in the coming years and presents new cohort characteristic data. The richness of the longitudinal data and biobank together with this manuscript may invite collaborative research and further examination of the genetic and epigenetic effects of glucose dysregulation.

Specific comments and suggestions may be found below.

Abstract & Introduction.
Line 62. Pluralize “limitations” and include sample size issues, as discussed in the Perspective section. i.e sample size is small for genetic variant studies, but reasonable for omics studies.
Line 103. Would be helpful to integrate important parts of the STROBE checklist in the introduction, to support the value of the cohort.

Methods.
Line 135. Why were buffy coats selected for the genetic component rather than buccal cells or other biosamples?
Line 141 & 146. Suggest citation to previous cohort paper with tools and techniques, or reference to methods later in the text.
Line 151. Why was the cutoff at age 3.5? The reconsent and decline percentages are helpful.
Line 157. Would this information be useful in a table to show which measurements were taken for mothers and children at years 3 and 5? Maybe as a supplemental table for researchers interested in using Gen3G data. Or, include references to tables in the text, e.g. Table 5.
A number of topics should also be included in the methods, such as cutoffs for GDM or diabetes diagnosis, whether socioeconomic confounders were assessed (income, education, employment, etc.)

Findings to Date.
Line 311-323. The summary of previous findings is helpful to researchers reading this update and interested in earlier Gen3G work.
Line 354. Suggest revising the use of “post-partum” visits in reference to child age throughout the manuscript.
Line 365. For the additional glucose tolerance testing, were pregnant mothers excluded? Was the mother’s pregnancy status assessed during followup visits?
Line 372. Suggest emphasizing cohort’s value among omics studies with long-term followup earlier in the manuscript.

Minor Comments:
Line 48, typo in Achievements to date
Line 53, missing “the” in “Using Gen3G rich dataset.
Line 62, typo in “Strengths”.
Line 169, typo resulting in a split sentence: “...provided kits. Which were returned by mail.”
Line 350, typo in “Strengths”
Line 384, typo in “microbiota”

**Reviewer**	**2**
**Name**	**Morcillo, Sonsoles**
**Affiliation**	**Instituto de Investigacion Biomedica de Malaga**

**Date**	**13-Nov-2024**
**COI**	**None**

The manuscript is clear, well written and provides a detailed and thorough description of the Gen3G cohort of pregnant women and their offspring, a well-established and widely recognized cohort that has contributed significantly to scientific advancements in understanding the impact of altered glucose regulation during pregnancy on maternal and offspring health, both in the short and long term. This cohort includes a huge amount of phenotypic, metabolic, anthropometric, lifestyle and “omic” information providing invaluable insights into mechanisms and potential biomarkers linked to future disease risk.

However, there are some issues that require clarification from the authors.

1. **Purpose:** While the article does an excellent job describing the cohort, the purpose of presenting this cohort study remains unclear. Clarifying the objective—such as encouraging consortia development, highlighting the importance of expanding research in this field, or underscoring the potential impact of further exploration—would strengthen the manuscript’s relevance beyond mere description.
2. Pag 3 of 41: the table presents intra- and inter-assay %CV for several parameters, but there isn’t any legend of figure along the manuscript referencing this table.
3. Page 10 of 41. Abstract: line 45 mentions “among the 695 mother-child dyads”. The origin of this specific number is not clear from the context provided. To improve clarity, it would be helpful to briefly explain within the abstract where this sample size comes from or how it was determined.
4. Study procedures (line 135): How many mothers agreed to participate in the genetic component of the study? Authors could Include this figure providing more context on sample representation.
5. Table 1: Table 1 shows no significant demographic differences between groups at baseline, which is valuable. However, it’s noteworthy that a higher proportion of women with GDM participated in the study compared to those who declined. This could inform future strategies for improving participation in follow-up studies, particularly among control (non-diseased) populations.
6. Regarding the questionnaires used throughout the study and in the various follow-up visits, I suggest specifying all questionnaires employed in Box 1. While some are indicated, such as the CES-D scale for depressive state, others remain unclear. For instance, which questionnaire was used to assess diet? How was the physical activity of the offspring, as reported by mothers, evaluated? It is important to specify all questionnaires used in each case. Additionally, Table 2 mentions the use of the CCHS, but it has not been previously explained what information it includes. Finally, concerning the "Questionnaire other health conditions" used for parents, I would appreciate it if you could specify which questionnaire was employed.
7. Could authors clarify if maternal BMI was collected pre-pregnancy? This information is relevant for later analyses (i.e., whether obesity was a pre-existing

risk factor). I notice, however, that BMI data were collected during the 3- and 5-year follow-ups.

8. Information regarding pharmacological treatment for mothers during pregnancy, such as insulin use or diet-based interventions, is missing. This data would be relevant for future analyses. I understand that this information is collected and described in previous articles, but it could be included in this article.
9. Table 2 presents *Medical history (self-reported as diagnosis*) from mothers. But it shows that no cases of type 2 diabetes were self-reported at 3 years and only one case at 5 years. Were any glucose regulation disorders diagnosed during follow-up visits? Mentioning this in the text would be appreciated.
10. The authors could explain the purpose of conducting the 75-g OGTT at the 5-yearfollow-up, but not at the 3-year. It could be explained that the choice to measure at 5 years may be based on the increased likelihood of detecting glucose metabolism alterations. Additionally, was insulin at different points of OGTT measured? Table 3 only shows “fasting insulin” in *Glycaemic regulation* variables.
11. Table 3 shows that only 143 saliva samples were collected at the 3-year follow-up, despite the participation of 448 mother-child pairs. Could the authors clarify the reason for this discrepancy in sample numbers?
12. Pag 29 of 41 (lines 275-276): Please indicate how blood samples were processed for future RNA extractions and how they were preserved, similar to the information provided for DNA.
13. The authors mention that they have collected hair samples from the children to measure cortisol levels. Do they plan to perform additional assessments using this type of sample?
14. One of the main challenges in longitudinal studies is participant adherence. There is often a high percentage of participants who do not attend follow-up visits, which can significantly impact the study. In this cohort, it appears that the participation rate in follow-up visits is around 70%, which is a good figure. The authors could include a paragraph, perhaps in the study limitations section, suggesting strategies to improve this aspect in future studies.
15. Additionally, it would be valuable to include a final reflection calling for an increase in this type of research focused on the DOHaD (Developmental Origins of Health and Disease) hypothesis. This reflection should emphasize the need to create consortia and foster collaborations to increase sample sizes, conduct comprehensive longitudinal studies, and replicate and validate findings across different populations.

**Reviewer**	**3**
**Name**	**Barrozo , Enrico**
**Affiliation**	**Baylor College of Medicine, Obstetrics and Gynecology**
**Date**	**15-Nov-2024**
**COI**	**None**

In the manuscript entitled, “Cohort profile: The Genetics of Glucose regulation in Gestation and Growth (Gen3G) - a prospective prebirth cohort of mother–child pairs in Sherbrooke, Canada, 3- and 5-year post-partum follow-ups” by Amélie Taschereau, Myriam Doyon, Mélina Arguin, Catherine Allard, Véronique Desgagné, Éric Massé, Pierre-Étienne Jacques, Patrice Perron, Anne-Marie Côté, Marie-France Hivert, and Luigi Bouchard, the authors describe the methodology, cohort characteristics, and previously published sequencing data from the Gen3G maternal-offspring participants that returned for longitudinal studies 3 and 5-years after delivery. The objectives of Gen3G were to investigate maternal metabolic health after delivery longitudinally and to understand how gestational metabolic exposures associated with obesity and neurodevelopmental problems in early childhood were potentially associated with molecular mechanisms of the Developmental Origins of Health and Disease (DOHaD). In addition, this manuscript reports on the progress the Gen3G has made towards achieving these objectives.

Overall, the strengths of this study include the multi-layered rigor and reproducibility in their approach and analysis, data presented in visually appealing tables, and thoughtful, critical writing. The sample sizes are consistent and accurate throughout.

The major weakness of this manuscript was a lack of novel findings, considering there already are >75 publications describing Gen3G. With enrollment ending in 2013, the 3 and 5-year follow-ups would have been completed by 2016 and 2019, respectively. In addition, the manuscript could be improved by discussing their findings in the context of over 30 years of research since the DOHaD hypothesis was first proposed.

Below are items the authors may consider addressing.

Major:

1. Consider including significant findings from the 3 and 5 year follow-ups beyond differences in the smoker and GDM participant follow-up rates

2. Lines 86-88: Cite Barker for the DOHaD hypothesis

3. Lines 86-88: Reference 7- there have been many advancements in the field since 2007

4. Line 306: All 75 publications should be cited

5. To improve the contribution to the literature, discuss the findings of the previous Gen3G studies in the introduction. Discuss the results from Gen3G in the context of other longitudinal studies in humans

6. Since epigenetic mechanisms were raised, discuss Gen3G epigenetic findings in the context of other human and/or animal models

Minor:

1. Minor typos and grammatical errors throughout- e.g., lines 40, 48, 62, 65, 74, 88, etc.

2. Figure 1: Capital N for the total population, likely N=1,024 enrolled. Lowercase n= for all subsequent sample sizes

3. Lines 356-359: Provide references supporting the statement about AI

**Reviewer**	**4**
**Name**	**Ouidir, Marion**
**Affiliation**	**Universite Grenoble Alpes**
**Date**	**21-Nov-2024**
**COI**	**None**

The paper presented is a cohort profile paper that describes the Genetics of Glucose Regulation in Gestation and Growth (Gen3G) prospective mother-child cohort conducted in Sherbrooke, Canada. It is well-written, with a clear presentation of the biological samples collected and a concise overview of some results.

Major comments:

- Could the authors provide more details about the hormones to be investigated in the 6th follow-up (line 384)?

- Were home addresses or residential histories collected during previous follow-ups? This information would be valuable for assessing environmental exposures.

- The biological samples collected make this cohort highly valuable to the research community. However, the paper does not clearly specify which biological samples were used for which measurements. For example, were urine samples analyzed? If so, for what purposes?

- The presentation of biological samples and data could be improved for clarity. It might be easier to follow if the biological samples collected (Table 5) are presented before the biological data measured (Table 3). Additionally, there is an apparent mismatch between the numbers of urine or stool samples reported in Tables 3 and 5. Could the authors explain this discrepancy?

Minor comments:

- In the discussion of future perspectives, could the authors specify whether both mothers and children will be included in the 6th follow-up?

- Please ensure consistency in the tables when presenting biological and anthropometric measurements. Use either “mean ± SD” or “median [IQR]” throughout the manuscript.

- For Table 4, it would be clearer to use only “median [IQR]” and update the header to remove “N(%)” since it is not applicable.

VERSION 1 - AUTHOR RESPONSE

Response to reviewer 1 comments

Abstract & Introduction.
Line 62. Pluralize "limitations" and include sample size issues, as discussed in the Perspective section. i.e sample size is small for genetic variant studies, but reasonable for omics studies.

Answer #1: As suggested we have included sample size issue in the limitations of our study (p. 4, L.74): "Sample size is relatively small for genetic association studies but reasonable for other 'omics studies in the context of refined phenotype and longitudinal follow-up of participants."

Line 103. Would be helpful to integrate important parts of the STROBE checklist in the introduction, to support the value of the cohort.

Answer #2: We are sorry, but this comment remains unclear since the introduction already includes all the items on the STROBE checklist: "The scientific background and rationale for the investigation being reported" and the "specific objectives, including any prespecified hypotheses." We will be happy to address this comment if clarified by the reviewer.

Methods.
Line 135. Why were buffy coats selected for the genetic component rather than buccal cells or other biosamples?

Answer #3: As requested, the reason to have selected the buffy coat for genetic analyses in Gen3G is now reported (p. 8, L.148): "We collected buffy coats for the genetic component of the study because they are easy to collect and provide a high quantity and quality of DNA [11]."

Line 141 & 146. Suggest citation to previous cohort paper with tools and techniques, or reference to methods later in the text.

Answer #4: As suggested, we have referred the standardized procedure to the method described later in the text, in the section on data collection (p. 8, L.154): "(all described in the data collection section)".

Line 151. Why was the cutoff at age 3.5? The reconsent and decline percentages are helpful.

Answer #5: Thanks for giving us the opportunity to clarify our recruitment strategy for these two visits relatively close to each other. This cut-off point was set to avoid overburdening mother-child dyads by requiring them to attend two visits within a year or so. To ensure

clarity, we have added the reason in the text (p. 8, L.165): "(cut-off point set to avoid overburdening mother-child dyads by requiring them to attend two visits too close together.)"

Line 157. Would this information be useful in a table to show which measurements were taken for mothers and children at years 3 and 5? Maybe as a supplemental table for researchers interested in using Gen3G data. Or, include references to tables in the text, e.g. Table 5.

Answer #6: We agree with the reviewer that reporting the measurements taken at the 3- and 5-years follow-up visits is needed. These can be found in tables 2, 3, 5 and 6, which we have preferred to present in the Data Collection Section of the manuscript. We feel it make more sense in that section than in the Study Procedures Section as suggested.

A number of topics should also be included in the methods, such as cutoffs for GDM or diabetes diagnosis, whether socioeconomic confounders were assessed (income, education, employment, etc.)

Answer #7: We thank this reviewer for his/her comment and for the opportunity to add precision in the method of our article.

1. GDM diagnosis: GDM diagnosis was based on IADPSG criteria. To add precision, we have defined the IADPSG cut-off points for GDM diagnosis in the text (p. 7, L.128): "(fasting plasma glucose ≥5.1 mmol/L and/or 1 h plasma glucose ≥10.0 mmol/L and/or 2 h plasma glucose ≥8.5 mmol/L)", as well as in the note under Table 1: "*By IADPSG criteria: 75g-OGTT with fasting, 1 and 2 h plasma glucose. If fasting plasma glucose ≥5.1 mmol/L and/or 1 h plasma glucose ≥10.0 mmol/L and/or 2 h plasma glucose ≥8.5 mmol/L."
2. Diabetes diagnosis: As suggested by the reviewer #2, we have added the number of cases of prediabetes and diabetes diagnosed at the 3- and 5-year follow-up. As suggested by this reviewer, we have added the cut-off points for diagnosis based on the American Diabetes Association criteria (p. 17, L.238): "Using American Diabetes Association (ADA) criteria [12], we identified 72 additional women with prediabetes (ADA criteria: fasting plasma glucose 5.6 mmol/L to 6.9 mmol/L and/or 2 h plasma glucose 7.8 mmol/L to 11.0 mmol/L and/or HbA1c 5.7% to 6.4%) and 4 additional women with type 2 diabetes (T2D) (ADA criteria: fasting plasma glucose ≥7 mmol/L and/or 2 h plasma glucose ≥11.1 mmol/L and/or HbA1c ≥6.5%) at the 3- and 5-years follow-up visits"
3. Socio-economic variables: We have also specified in the text which socio-economic variables were collected during the follow-up visits (p. 8, L153): "(including educational level and current marital status)".

Findings to Date.
Line 311-323. The summary of previous findings is helpful to researchers reading this update and interested in earlier Gen3G work.
Answer: Thank you

Line 354. Suggest revising the use of "post-partum" visits in reference to child age throughout the manuscript.

Answer #8: As suggested by this reviewer, we have revised the entire manuscript to change the use of 'post-partum' visit in relation to the age of the child. Specifically, the two visits originally referred to as the 3- and 5-years post-partum visits have been renamed as the 3- and 5-years follow-up visits.

Line 365. For the additional glucose tolerance testing, were pregnant mothers excluded? Was the mother's pregnancy status assessed during followup visits?

Answer #9: Pregnancy status was assessed at the 3-year and 5-year follow-up visits and pregnant women were excluded from the additional glucose tolerance testing, blood draws and anthropometric measurements. In the revised version of the manuscript, we have added this information in the text (p. 10, L.196): "At both 3- and 5-years follow-up visits, we assessed maternal pregnancy status and pregnant women did not undergo anthropometric measurements, blood sampling and the 75g OGTT (5-year visit)." We have also added the number of pregnant women for each follow-up visits in table 2.

Line 372. Suggest emphasizing cohort's value among omics studies with long-term follow-up earlier in the manuscript.

Answer #10: As suggested by this reviewer, we highlighted the value of the study including omics data with longitudinal follow-up earlier in the manuscript, in the Introduction (p. 5, L.96): "Second, with this longitudinal follow-up, we aimed to expand our understanding of gestational metabolic exposures associated with obesity and neurodevelopmental problems in early childhood. Using 'omics analyses, we aimed to identify potential underlying molecular mechanisms of DOHaD."

Minor Comments:
Line 48, typo in Achievements to date
Line 53, missing "the" in "Using Gen3G rich dataset.
Line 62, typo in "Strengths".
Line 169, typo resulting in a split sentence: "…provided kits. Which were returned by mail."
Line 350, typo in "Strengths"
Line 384, typo in "microbiota"

Answer #11: We would like to thank this reviewer for pointing out the typographical errors, which have all been corrected in the revised version of the manuscript.

Response to reviewer 2 comments

1. **Purpose:** While the article does an excellent job describing the cohort, the purpose of presenting this cohort study remains unclear. Clarifying the objective—such as encouraging consortia development, highlighting the importance of expanding

research in this field, or underscoring the potential impact of further exploration—would strengthen the manuscript's relevance beyond mere description.

Answer #1: As suggested by this reviewer, we have now clarified the aim of presenting this study in the introduction (p. 6, L.116): "By presenting this cohort profile, we aimed to share the methods and procedures we have developed for monitoring mothers and children from the first weeks of pregnancy and now up to 5 years after birth, and to encourage collaboration with other cohorts, and the use of the Gen3G Cohort data and biosamples."

2. Page 23 of 41: the table presents intra- and inter-assay %CV for several parameters, but there isn't any legend of figure along the manuscript referencing this table.

Answer #2: The supplementary table 1 reporting intra- and inter-assay %CV for the biomarkers we have measured is referenced in page 26. Assessing and reporting intra- and inter-assay %CV provides the overall reliability of the assay is part of good laboratory practices.

3. Page 10 of 41. Abstract: line 45 mentions "among the 695 mother-child dyads". The origin of this specific number is not clear from the context provided. To improve clarity, it would be helpful to briefly explain within the abstract where this sample size comes from or how it was determined.

Answer #3: As suggested and to improve clarity, we have briefly explained in the revised version of the manuscript where the sample size comes from and how it was determined (p. 3, L.45): "Of the 1024 women originally recruited in the first trimester of pregnancy, we have targeted the 854 who had complete glucose tolerance test data and the 724 newborns who provided placenta and/or cord blood samples for follow-up recruitment. Of these, 695 mother-child dyads agreed to be contacted for the prospective follow-up visits."

4. Study procedures (line 135): How many mothers agreed to participate in the genetic component of the study? Authors could Include this figure providing more context on sample representation.

Answer #4: As suggested we have indicated the number of mothers who agreed to participate in the genetic component of the study for themselves (p. 8, L.147) and for their children (p. 9, L.180).

5. Table 1: Table 1 shows no significant demographic differences between groups at baseline, which is valuable. However, it's noteworthy that a higher proportion of women with GDM participated in the study compared to those who declined. This could inform future strategies for improving participation in follow-up studies, particularly among control (nondiseased) populations.

Answer #5: This reviewer is right, a higher proportion of women with GDM participated at the 3- and 5-years follow-up visits. We already report in the manuscript (p. 10, L.191) that "17 women with GDM or low birth weight, for whom we did not collect a biological sample at birth and who were excluded from the 3y follow-up initially, were invited to the 5-year follow-up visit." This may have contributed to a higher proportion of GDM in the study group for this follow-up (5 years). Also, women with GDM might have felt more involved in the study, as we have originally explained in the consent form that GDM was one of the main interest of the cohort. The recruitment strategy for both follow-up visits (3 and 5 years) was not different for women with and without GDM.

6. Regarding the questionnaires used throughout the study and in the various follow-up visits, I suggest specifying all questionnaires employed in Box 1. While some are indicated, such as the CES-D scale for depressive state, others remain unclear. For instance, which questionnaire was used to assess diet? How was the physical activity of the offspring, as reported by mothers, evaluated? It is important to specify all questionnaires used in each case. Additionally, Table 2 mentions the use of the CCHS, but it has not been previously explained what information it includes. Finally, concerning the "Questionnaire other health conditions" used for parents, I would appreciate it if you could specify which questionnaire was employed.

Answer #6: Maternal and offspring diet and physical activity were assessed using an adapted version of the Canadian Community Health Survey (CCHS). As suggested, a reference to this questionnaire has been added to the text just before it is mentioned in Table 2 (p. 8, L.150): "At the 3-year follow-up visit, we collected mothers' medical history and lifestyle habits (nutrition and physical activity using an adapted version of the Canadian Community Health Survey (CCHS), alcohol consumption, smoking status, sleep duration, and sun exposure)" and children (p. 8, L.155): "We asked the mothers about their child's medical history and lifestyle habits (nutrition and physical activity using an adapted version of the CCHS, sleep duration, second-hand smoke exposed and screen time)". This questionnaire has also been clarified in Box 1. Concerning the "questionnaire on other health conditions" used for parents, no questionnaire was employed, it was simply an open question. We rephrase the name of this variable as "Other health conditions (N available)" to avoid confusion (Table 2, p. 15).

7. Could authors clarify if maternal BMI was collected pre-pregnancy? This information is relevant for later analyses (i.e., whether obesity was a preexisting risk factor). I notice, however, that BMI data were collected during the 3- and 5-year follow-ups.

Answer #7: The Gen3G longitudinal prospective antenatal cohort was initiated in 2010 (Sherbrooke) and recruited 1024 women in their first trimester of pregnancy. The pregnancy phase of the cohort has already been described in a previous publication entitled: "Genetics of Glucose regulation in Gestation and Growth (Gen3G): a prospective prebirth cohort of mother–child pairs in Sherbrooke, Canada." (https://doi.org/10.1136/bmjopen-2015-010031). All information on pre-pregnancy maternal variables is reported in this publication. For the interest of this reviewer, pre-pregnancy BMI was calculated using the mother's self-

reported weight (kg) and height (m), which is a valid and recommended way to measure pre-gestational BMI (10.1016/0021-9681(82)90085-6).

8. Information regarding pharmacological treatment for mothers during pregnancy, such as insulin use or diet-based interventions, is missing. This data would be relevant for future analyses. I understand that this information is collected and described in previous articles, but it could be included in this article.

Answer #8: As suggest by this reviewer, we have added the number of women with GDM treated with insulin in Table 1.

9. Table 2 presents *Medical history (self-reported as diagnosis*) from mothers. But it shows that no cases of type 2 diabetes were self-reported at 3 years and only one case at 5 years. Were any glucose regulation disorders diagnosed during follow-up visits? Mentioning this in the text would be appreciated.

Answer #9: As suggested, we have added the number of cases of prediabetes and diabetes diagnosed at the 3 and 5- years post-partum follow-up (p. 17, L.238): "Using American Diabetes Association (ADA) criteria [12], we identified 72 additional women with prediabetes (ADA criteria: fasting plasma glucose 5.6 mmol/L to 6.9 mmol/L and/or 2 h plasma glucose 7.8 mmol/L to 11.0 mmol/L and/or HbA1c 5.7% to 6.4%) and 4 additional women with type 2 diabetes (T2D) (ADA criteria: fasting plasma glucose ≥7 mmol/L and/or 2 h plasma glucose ≥11.1 mmol/L and/or HbA1c ≥6.5%) at the 3- and 5-years follow-up visits."

10. The authors could explain the purpose of conducting the 75-g OGTT at the 5-yearfollow-up, but not at the 3-year. It could be explained that the choice to measure at 5 years may be based on the increased likelihood of detecting glucose metabolism alterations. Additionally, was insulin at different points of OGTT measured? Table 3 only shows "fasting insulin" in *Glycaemic regulation* variables.

Answer #10:

As the two prospective childhood follow-up visits were relatively close to each other (3 and 5 years of age) and the OGTT was a relatively long, difficult and costly test to administer, we decided it would be included only at the 5-year follow-up visit.

Insulin levels were also measured at different time points during the OGTT. We have added the insulin measurements at 1h and 2h post-OGTT in Table 5 (originally Table 3).

11. Table 3 shows that only 143 saliva samples were collected at the 3-year follow-up, despite the participation of 448 mother-child pairs. Could the authors clarify the reason for this discrepancy in sample numbers?

Answer #11: Saliva sample collection as an ancillary study was added to the protocol after the 3-year follow-up visit recruitment started. The note of the Table 6 (originally Table 3) was clarified to this end.

12. Pag 19 of 41 (lines 275-276): Please indicate how blood samples were processed for future RNA extractions and how they were preserved, similar to the information provided for DNA.

Answer #12: As suggested by the reviewer, we have indicated how blood samples were processed for future RNA extractions (p. 25, L.311): "Plasma aliquots of ˜500 µl were wrapped in parafilm to avoid evaporation and stored at -80°C for future RNA extraction and analysis or biobanking (Table 4)."

13. The authors mention that they have collected hair samples from the children to measure cortisol levels. Do they plan to perform additional assessments using this type of sample?

Answer #13: No further analysis of the hair samples is planned at this time but some samples remain available in our Gen3G biobank.

14. One of the main challenges in longitudinal studies is participant adherence. There is often a high percentage of participants who do not attend follow-up visits, which can significantly impact the study. In this cohort, it appears that the participation rate in follow-up visits is around 70%, which is a good figure. The authors could include a paragraph, perhaps in the study limitations section, suggesting strategies to improve this aspect in future studies.

Answer #14: As suggested, we have included a paragraph in the study strengths and limitations section explaining the strategies used to encourage participation in our study (p. 29, L.411): "Another strength of our study is the high retention rate of participants over the first 6-7 years of the study. Over 70% of the families who agreed to be contacted returned for the 3- and 5-years follow-up visits. We used several strategies to optimize follow-up participation to the study, including stable research staff, sending annual Christmas cards to keep participants updated on the progress of the project, and disseminating research findings in layman's terms via social media. We updated participants' contact details at each follow-up visit. Staff invited participants for follow-up visits during the school holidays (e.g. summer vacations, spring breaks) and over the weekend days. We augmented research staff and trainees during the summer vacations to provide additional visits opportunities while children were not in school. Finally, for women who were unable to come to the research center in person, we offered the opportunity to complete the questionnaires remotely, and women who were able to come in person but had limited time, we offered shorter visits with fewer components (and the option of completing the questionnaires remotely)."

15. Additionally, it would be valuable to include a final reflection calling for an increase in this type of research focused on the DOHaD (Developmental Origins of Health and Disease) hypothesis. This reflection should emphasize the need to create consortia and foster collaborations to increase sample sizes, conduct comprehensive longitudinal studies, and replicate and validate findings across different populations.

Answer #15: As suggested, we have added a reflection on the relevance of this type of longitudinal study to elucidate DOHAD mechanisms (p. 28, L.388): "Longitudinal cohort studies such as Gen3G are critical to elucidate the mechanisms supporting the DOHAD hypothesis in human, which would help to develop novel prevention strategies. They also contribute to foster collaborations between research teams and international consortia to create larger datasets, conduct meta-analyses and validate results in diverse populations."

Response to reviewer 3 comments

Below are items the authors may consider addressing.
Major:
1. Consider including significant findings from the 3 and 5 year follow-ups beyond differences in the smoker and GDM participant follow-up rates

Answer #1: We are sorry, but this comment is unclear to the authors. We would nevertheless like to draw the reviewer's attention to the fact that Gen3G's main findings from publications over the last 10 years is provided in the Discussion Section. We hope this adequately answers this comment.

2. Lines 86-88: Cite Barker for the DOHaD hypothesis

Answer #2: As suggested, we have cited Barker for DOHaD hypothesis (p. 5, L.87): According to the Developmental Origin of Health and Diseases (DOHaD) hypothesis, derived from Barker's hypothesis [7], these predispositions might be programmed by epigenetics modifications [8], but the pathophysiological mechanisms supporting the DOHaD remain largely unknown.

3. Lines 86-88: Reference 7- there have been many advancements in the field since 2007

Answer #3: We agree with this reviewer and have added a more recent reference to this statement.

4. Line 306: All 75 publications should be cited

Answer #4: As suggested, the publications to which Gen3G data contributed are now reported in Supplementary table 2.

5. To improve the contribution to the literature, discuss the findings of the previous Gen3G studies in the introduction. Discuss the results from Gen3G in the context of other longitudinal studies in humans.

Answer #5:

1. As suggested, we have added a sentence in the Introduction to introduce Gen3G findings over time (p. 6, L.110): "Gen3G has contributed to the development of scientific knowledge in four major domains: 1- pathophysiology of glucose regulation in pregnancy and heterogeneity of GDM; 2- epidemiologic analyses supporting the DOHaD hypothesis; 3- potential mechanisms implicated in DOHaD, specifically epigenetic investigations, and 4- contributions to large genetic and epigenetic consortia related to pregnancy and early life metabolic traits. We provided an overview of our contributions in the discussion of this cohort profile." More details are provided, in the discussion Section
2. As suggested, we have added a sentence at the end of the discussion to contextualize the strengths of the Gen3G from other longitudinal pregnancy cohort studies in North America (p. 28, L.381): "Gen3G is not the only longitudinal mother-child cohort that started in pregnancy and prospectively follow-up participants in childhood. Some other well-known cohorts with similar aims and data include Project Viva, ALSPAC, Generation R, iABC, FAMILY, and START. Gen3G set itself apart by its detailed phenotyping overall and glucose physiology in particular, its rich biobank and the assessment body fat using DEXA scan in children at 5 years of age. Our glycemic phenotype in pregnancy, and rich 'omics data makes Gen3G similar in many ways to the HAPO follow-up study, with whom we have collaborated multiple times to replicate each other's findings, adding to the robustness of our publications."

6. Since epigenetic mechanisms were raised, discuss Gen3G epigenetic findings in the context of other human and/or animal models

Answer #6: Gen3G epigenetic findings were summarized in pages 26-28.

Minor:
1. Minor typos and grammatical errors throughout- e.g., lines 40, 48, 62, 65, 74, 88, etc.

Answer #7: All typographical errors have been corrected. The spelling of the entire article has been revised.

2. Figure 1: Capital N for the total population, likely N=1,024 enrolled. Lowercase n= for all subsequent sample sizes

Answer #8: The figure has been changed in line with this comment.

3. Lines 356-359: Provide references supporting the statement about AI

Answer #9: We have added a reference to support the statement on AI and its future application in reproductive and precision medicine.

Response to reviewer 4 comments

Major comments:
- Could the authors provide more details about the hormones to be investigated in the 6th follow-up (line 384)?

Answer #1: As suggested by this reviewer, we have added this information to the manuscript (p. 30, L.438): "The overarching goal of the 6th reassessment is to study how prenatal and early-life factors program childhood adiposity and obesogenic dietary behaviors, and to investigate peripheral signals (e.g. microbiota and hormones, including insulin and adipokines) contributing to central (hypothalamus) regulation of childhood adiposity and dietary behaviors."We intend to measure more hormones and biomarkers, but funding is still lacking.

- Were home addresses or residential histories collected during previous follow-ups? This information would be valuable for assessing environmental exposures.

Answer #2: Unfortunately, home addresses or residential histories were not collected at the previous follow-up, nor at the 3- and 5-year follow-ups.

- The biological samples collected make this cohort highly valuable to the research community. However, the paper does not clearly specify which biological samples were used for which measurements. For example, were urine samples analyzed? If so, for what purposes?

Answer #3: We collected urine samples for assessment of albuminuria and podocyturia. This has been clarified in the text (p. 29, L.405): "We collected urine samples for assessment of albuminuria and podocyturia to monitor renal function and stool samples for assessment of gut microbiota, which is of interest since microbiota disruption has been associated with the development of T2D [71]."

- The presentation of biological samples and data could be improved for clarity. It might be easier to follow if the biological samples collected (Table 5) are presented before the biological data measured (Table 3). Additionally, there is an apparent mismatch between the numbers of urine or stool samples reported in Tables 3 and 5. Could the authors explain this discrepancy?

Answer #4:

1. As suggested, we have reorganized the text to present the table "Biological samples collected at the 3- and 5-years follow-up visits" before the tables "Biological data from Gen3G mothers collected during the 3- and 5-years follow-up visits" and "Biological data from Gen3G children collected during the 3- and 5-years follow-up visits" (originally the table "Gen3G participant's biological data collected during the 3- and 5-years follow-up visits.") The section "Anthropometry and vital signs (3-year and 5-year)" has been moved after the section "Blood and other biological samples collected (3-year and 5-year)" to ensure consistency in the order of data presentation in the text and tables.

2. Table 4 in the revised version of the manuscript (previously Table 5), refers to the number of participants who accepted biobanking of these biological samples. All participants that consented to data collection (with or without consent for biosamples biobanking and sharing) are reported in new Tables 5 and 6 in the revised version of the manuscript (previously Table 3).

Minor comments:
- In the discussion of future perspectives, could the authors specify whether both mothers and children will be included in the 6th follow-up?

Answer #5: As suggested, we have clarified that both mothers and children are invited in the 6th follow-up (p. 30, L.432): "Gen3G recently engaged in its 6th follow-up, 10-12 years after birth, and we are inviting both the mother and the children to participate."

- Please ensure consistency in the tables when presenting biological and anthropometric measurements. Use either "mean ± SD" or "median [IQR]" throughout the manuscript.

Answer #6: We understand the comment of this reviewer, but we have adhered to the guidelines, which state that means ± SD should be reported for normally distributed data and median ± IQR for non-normally distributed data.

- For Table 4, it would be clearer to use only "median [IQR]" and update the header to remove "N(%)" since it is not applicable.

Answer #7: Thanks for pointing out the inaccuracy in table 4. We have removed "N(%)" since it was not applicable.

VERSION 2 - REVIEW

**Reviewer**	**2**
**Name**	**Morcillo, Sonsoles**
**Affiliation**	**Instituto de Investigacion Biomedica de Malaga**
**Date**	**21-Jan-2025**
**COI**	

The authors have addressed all the questions, and the manuscript has improved significantly. I appreciate the effort made by the authors to respond to all my questions.

I am intrigued by the choice of plasma as the sample type for future RNA isolation, instead of using PBMCs separated by Ficoll density gradient, for example. Plasma is commonly used for microRNA isolation. Could the authors please clarify if the focus of this study is

specifically on microRNAs, or if there is another rationale behind selecting plasma for RNA isolation?

VERSION 2 - AUTHOR RESPONSE

Response to reviewer 2 comments

The authors have addressed all the questions, and the manuscript has improved significantly. I appreciate the effort made by the authors to respond to all my questions.

Thank you

I am intrigued by the choice of plasma as the sample type for future RNA isolation, instead of using PBMCs separated by Ficoll density gradient, for example. Plasma is commonly used for microRNA isolation. Could the authors please clarify if the focus of this study is specifically on microRNAs, or if there is another rationale behind selecting plasma for RNA isolation?

Answer 1: This reviewer is correct, we used plasma rather than PBMCs for RNA isolation because the focus of this study was to find biomarkers such as microRNAs, but also because plasma is easier to collect and less time consuming than PBMCs, which require additional steps to isolate. This is important in the context of our study, where samples were collected with limited time and research staff, during experimental pregnancy visits, including several other assessments.
